# Mitochondrial DNA methylation in metabolic associated fatty liver disease

**DOI:** 10.3389/fnut.2023.964337

**Published:** 2023-05-25

**Authors:** Archibold Mposhi, Fabian Cortés-Mancera, Janette Heegsma, Vincent E. de Meijer, Bart van de Sluis, Svenja Sydor, Lars P. Bechmann, Claudia Theys, Peter de Rijk, Tim De Pooter, Wim Vanden Berghe, İkbal Agah İnce, Klaas Nico Faber, Marianne G. Rots

**Affiliations:** ^1^Department of Pathology and Medical Biology, University of Groningen, University Medical Center Groningen, Groningen, Netherlands; ^2^Department of Gastroenterology and Hepatology, University of Groningen, University Medical Center Groningen, Groningen, Netherlands; ^3^Departamento de Ciencias Aplicadas, Instituto Tecnológico Metropolitano, Medellín, Colombia; ^4^Department of Surgery, Division of Hepato-Pancreato-Biliary Surgery and Liver Transplantation, University of Groningen, University Medical Center Groningen, Groningen, Netherlands; ^5^Section of Molecular Genetics, University of Groningen, University Medical Center Groningen, Groningen, Netherlands; ^6^Department of Internal Medicine, University Hospital Knappschaftskrankenhaus, Bochum, Germany; ^7^Ruhr-University Bochum, Bochum, Germany; ^8^Department of Biomedical Sciences, University of Antwerp, Antwerp, Belgium; ^9^Neuromics Support Facility, VIB-UAntwerp Center for Molecular Neurology, University of Antwerp, Antwerp, Belgium; ^10^Department of Medical Microbiology, School of Medicine, Acıbadem Mehmet Ali Aydınlar University, Istanbul, Türkiye

**Keywords:** liver steatosis, NAFLD, NASH, ND6, mtDNA methylation

## Abstract

**Introduction:**

Hepatic lipid accumulation and mitochondrial dysfunction are hallmarks of metabolic associated fatty liver disease (MAFLD), yet molecular parameters underlying MAFLD progression are not well understood. Differential methylation within the mitochondrial DNA (mtDNA) has been suggested to be associated with dysfunctional mitochondria, also during progression to Metabolic Steatohepatitis (MeSH). This study further investigates whether mtDNA methylation is associated with hepatic lipid accumulation and MAFLD.

**Methods:**

HepG2 cells were constructed to stably express mitochondria-targeted viral and prokaryotic cytosine DNA methyltransferases (mtM.CviPI or mtM.SssI for GpC or CpG methylation, respectively). A catalytically inactive variant (mtM.CviPI-Mut) was constructed as a control. Mouse and human patients’ samples were also investigated. mtDNA methylation was assessed by pyro- or nanopore sequencing.

**Results and discussion:**

Differentially induced mtDNA hypermethylation impaired mitochondrial gene expression and metabolic activity in HepG2-mtM.CviPI and HepG2-mtM.SssI cells and was associated with increased lipid accumulation, when compared to the controls. To test whether lipid accumulation causes mtDNA methylation, HepG2 cells were subjected to 1 or 2 weeks of fatty acid treatment, but no clear differences in mtDNA methylation were detected. In contrast, hepatic Nd6 mitochondrial gene body cytosine methylation and Nd6 gene expression were increased in mice fed a high-fat high cholesterol diet (HFC for 6 or 20 weeks), when compared to controls, while mtDNA content was unchanged. For patients with simple steatosis, a higher ND6 methylation was confirmed using Methylation Specific PCR, but no additional distinctive cytosines could be identified using pyrosequencing. This study warrants further investigation into a role for mtDNA methylation in promoting mitochondrial dysfunction and impaired lipid metabolism in MAFLD.

## Introduction

Mitochondria are the main cellular energy producers and key drivers of metabolism. Mitochondrial dysfunction results in a variety of (metabolic) diseases, including metabolic-associated fatty liver disease (MAFLD) (1, 2). Mitochondria contain their own circular genome (mtDNA), which is approximately 16.5 kb in size and encompasses 37 genes: 13 protein-coding genes, 2 ribosomal RNAs (rRNAs), 22 transfer RNAs (tRNAs) (3–5), non-coding RNAs including lncND5, lncCytb, lncND6 and lncRNAs with largely unknown functions (6).

The term MAFLD covers a wide spectrum of liver pathologies associated with fat accumulation, including simple steatosis (SS), metabolic steatohepatitis (MeSH), MeSH-associated fibrosis and cirrhosis, which are both risk factors for eventually developing hepatocellular carcinoma (HCC) (7). During MAFLD progression, mitochondria undergo structural and molecular changes that impair their function (8). The mitochondrial genome itself plays a role in the development and progression of MAFLD. For instance, individuals with mitochondrial haplogroup H share a common single nucleotide polymorphism in the mtDNA and are more susceptible to MeSH, while those with haplogroup L appear relatively protected against MeSH and fibrosis (9). With respect to differences in mtDNA content, seemingly contradictory data have been reported: In human liver samples, a higher mtDNA content was observed for SS and other early stages of MAFLD (10, 11), while a lower mtDNA content was reported in studies assessing patient cohorts that included cases with histologically MeSH-associated fibrosis (12, 13) compared to controls without fatty livers. These data underscore the dynamic nature of mitochondrial compensation mechanisms (10, 11, 14).

Decreased mitochondrial copy number is assumed to translate to reduced mitochondrial gene expression, but intricate details on how mitochondrial gene expression is regulated remain elusive (5, 15). The mtDNA contains a non-coding regulatory region, known as the displacement loop (D-loop), which contains the three promoter regions, namely HSP1 and HSP2 initiating transcription of the Heavy Strand and the LSP for the Light Strand (16). The HSP1 regulates transcription of *12S* and *16S* ribosomal RNAs, while the HSP2 promotes transcription of the entire H-strand as a polycistronic transcript containing 12 of the 13 protein-coding genes. The LSP regulates the transcription of the one protein-coding gene present on L-strand, the complex 1-subunit *ND6*, as well as eight tRNAs (16).

It has been suggested that epigenetic modifications on the mtDNA play a role in regulating the expression of mitochondrial genes. Indeed, we previously demonstrated that targeting DNA methyltransferases to the mitochondria induced mtDNA methylation, which repressed gene expression in a context-dependent manner (17). Methylation of mtDNA could serve as an adaptation to cellular stress that enables the mitochondria to function in stressed conditions. For instance, in the yeast *Candida albicans*, continuous exposure to hypoxic conditions decreased mtDNA methylation (18). Importantly, this phenomena has also been observed for mammalian mtDNA in response to external stress factors, such as air pollution (19, 20), exposure to arsenic-contaminated water (21) and high fat diet-induced insulin resistance, or upon treatment with AMPK activators (22). In fact, numerous studies indicated that differential mtDNA methylation associates with clinical phenotypes such as diabetes, colon cancer, neurodegenerative diseases, as well as in aging (22–24). Importantly, DNA methyltransferases (DNMTs) and the counteracting Ten-Eleven-Translocation (TET) enzymes have indeed been reported to be present in the mitochondria (25). Furthermore, modulating the expression of DNMTs by overexpression or gene-knock down resulted in increased and decreased mtDNA methylation, respectively (26). Of note, also for MAFLD, *Pirola and coworkers* demonstrated increased *ND6* methylation for liver samples from MeSH patients versus SS subjects, which related to a decrease in *ND6* gene expression (8).

Despite these intriguing indications that mtDNA methylation plays a biological role in cell pathophysiology, the actual existence of mtDNA methylation is still heavily debated (23, 27). This debate is in part fueled by the technical challenges in determining methylation in the tightly coiled mtDNA structures. For instance, techniques such as pyrosequencing depend on bisulfite conversion and are prone to bias if cytosine residues are shielded and therefore resistant to conversion. Indeed, the supercoiled structure of mtDNA prevents the complete conversion of unmethylated cytosines, which results in an overestimation of methylation (28–30). The supercoiled structure can be relaxed by fragmenting the mtDNA with restriction endonucleases or sonication, thereby improving bisulfite conversion efficiency (28, 29, 31). Importantly, however, differential mtDNA methylation is also detected when using bisulfite-independent techniques, such as mass spectrometry (32), although here, nuclear DNA contamination will influence the outcomes (24, 33). More recently, efforts using long-read sequencing technologies have attempted to overcome these technical issues, but conflicting outcomes remain (34–37).

For MAFLD, the relationship with mitochondrial dysfunction has been extensively described, although the exact mechanisms initiating SS and its subsequent progression to MeSH remain elusive. It is known that the development of MAFLD depends on a myriad of other factors that include obesity, insulin resistance, and genetic predisposition (38), as well as epigenetic factors (39, 40). Whether epigenetic changes in mtDNA can initiate mitochondrial dysfunction in the liver remains to be established, although some strong associations have recently been reported (22).

Here, we first set out to address the functional effects of mtDNA methylation in liver cells. We engineered HepG2 cells to express mitochondrial-targeted DNA methyltransferases and assessed the effect on cellular lipid accumulation, mitochondrial function and DNA methylation. Moreover, we determined the effect of lipid exposure on mtDNA methylation of wildtype HepG2 cells and analyzed livers of diet-induced MAFLD mice and patients. In sum, our findings support a potential role of mtDNA methylation in mitochondrial dysfunction and lipid accumulation, with alterations in mitochondrial gene expression and differential methylation levels on selected cytosine sites of *ND6* in mice and in patients with MAFLD.

## Materials and methods

### Cell and culture conditions

Human hepatocarcinoma cells (HepG2) (ATCC, Manas, VA, USA) were cultured in Dulbecco’s Modified Eagle Medium (DMEM) + GlutaMAX (Gibco, Carlsbad, CA, USA) supplemented with 1% Penicillin–Streptomycin-Fungizone (PSF) and 10% Fetal Calf Serum (FCS) (Lonza, Verviers, Belgium) at 37°C in a humidified 5% CO_2_ incubator. Human embryonic kidney cells, HEK293T (ATCC) were cultured in DMEM + GlutaMAX (Gibco) with similar supplementation to HepG2 cells. During transfection, DMEM was supplemented with 1% PSF and 5% FCS (Lonza) at 37°C in a humidified 5% CO_2_ incubator.

### Plasmids and constructs

Previously, mitochondria targeted mtM.CviPI, mtM.CviPI-Mut (catalytically inactive) and mtM.SssI were cloned in pCDH-CMV-MCS-SV40-puro plasmid (17). The resultant pCDH-CMV-master synthetic construct-conII-SV40-puro containing a mitochondrial localization signal (MLS), an HA-tagged-flexible linker, followed by [MSssI/ MCviPI/ MCviPI-Mut] and two nuclear export signals (NES) were subsequently used for transductions. HEK293T cells were seeded at 700,000 cells per well in a 6-wells plate for 16 h. After cells had reached 70%–80% confluency, polyethylenimine (PEI) (Sigma-Aldrich, St. Louis, USA) and plasmid DNA (pCDH-MSssI/ MCviPI/ MCviPI-Mut) were added at a volume to mass ratio of 1:4 (Supplementary Table S1). After 48 h, the medium containing virus particles was collected and filtered directly onto HepG2 cells using a 0.45 μmol/L millex HV PVDF filter (Merck Millipore, Darmstadt, Germany). To validate expression of the methyltransferase, qPCR was performed using primers that recognize the target sequences (Supplementary Figures S1B,C). Antibiotic selection was carried out on HepG2-mtM.CviPI, HepG2-mtM.CviPI-Mut, and HepG2-mtM.SssI using different concentrations of puromycin (1–4 μg/ml) (Supplementary Figure S1C).

### Animals

C57BL/6 J mice (Charles River, Saint-Germain-Nuelles, France) were age- and sex-matched (8–10 weeks old), and fed either regular chow or high fat, high cholesterol (HFC) diet containing 21% fat, with 45% calories from butter-fat and 0.2% cholesterol per gram of diet (Scientific Animal Food and Engineering (SAFE), Villemoisson-Sur-Orge, France) for 6 weeks (*n* = 6; 6wkHFC) or 20 weeks (*n* = 8; 20wkHFC) similar to earlier studies (41, 42). Animals were kept in a pathogen-free environment with alternating dark–light cycles of 12 h, controlled temperature (20°C–24°C) and relative humidity (55% ± 15%). 6wkHFC animals were housed in the animal facility of the Otto-von-Guericke University hospital Magdeburg according to the recommendations of the Federation of European Laboratory animals (FELASA). All procedures were approved by the Landesamt für Natur-, Umwelt-, und Verbraucherschutz Northrhine Westfalia (LANUV NRW) and the Landesverwaltungsamt Saxony-Anhalt (reference number: 84.0204.2013.A082). 20wkHFC animals were housed under standard laboratory conditions according to the Dutch law on the welfare of laboratory animals and guidelines of the ethics committee of the University of Groningen for the care and use of laboratory animals. Animals received food and water *ad libitum* and were fasted 4 h before termination. Tissues were snap-frozen in liquid nitrogen or fixed in paraformaldehyde.

### Human liver samples

Investigations in human material and the use of patient liver samples were approved by the Ethics Committee (Institutional Review Board) of the University Hospital Essen (Reference Number: 09–4252) and the study protocol conformed to the ethical guidelines of the Declaration of Helsinki. Sample allocation for patients that underwent bariatric surgery was undertaken following patients’ informed consent. Liver samples from eight patients and five healthy control individuals (without MAFLD) were collected during surgery.

### Free fatty acid preparation

Sodium palmitate (PA) (Sigma-Aldrich) and/or Sodium oleate (OA) (Sigma-Aldrich) were dissolved in phosphate buffered saline (PBS) (Gibco) and placed in a water bath for 1 h at 70°C. 10% fatty acid free bovine serum albumin (BSA) (Sigma-Aldrich) was dissolved separately in PBS at 37°C. 10 mmol/L stock solutions of PA/OA (molar ratio 1:2) and PA only were prepared by mixing the 10% BSA solution with the PA/OA solution at room temperature to allow for conjugation.

### Oil Red O staining (ORO)

ORO (Sigma-Aldrich, O-0625) was dissolved in 99% 2-propanol on a roller mixer overnight at room temperature. The solution was filtered using Whatman size 4 filter paper (Whatman International, Buckinghamshire, UK) and diluted with demi-water at a ratio of 2 parts water and 3 parts ORO solution. Prior to staining, cells were fixed with 4% Formaldehyde for 10 min. Cells were then rinsed with 60% isopropanol for 30 s, stained with ORO stain for 10 min, removed, and rinsed with 60% isopropanol for 5 s. Cells were rinsed with demi-water for approximately 1 min, and Mayer’s hematoxylin solution (Sigma) was added for 10 min. The cells were then rinsed twice for 30 s with demi-water, air-dried and mounted with Crystal/MountTM (Biomeda Corp, Foster City, CA, USA).

### RNA isolation and reverse transcriptase quantitative PCR (RT-qPCR)

Total RNA was extracted from HepG2 cell lines using Trizol (Sigma-Aldrich) and quantified using a Nanodrop 1000 spectrophotometer (Thermo Scientific, Waltham, MA, USA). 2.5 μg RNA was treated with DNaseI (Thermo Scientific) and reverse transcribed using random hexamer primers with M-MLV Reverse transcriptase to generate cDNA, according to the manufacturer’s protocol (Thermo Scientific). Each qRT-PCR reaction contained 10 μmol/L of the antisense and sense primers (Supplementary Table S2) (Sigma-Aldrich), 10 ng cDNA, and 2x ABsolute QPCR SYBR Green Rox Mix (Thermo Scientific). Real-Time qPCR was carried out on the ViiA7 Real-time PCR system (Applied Biosystems, Thermo Fisher Scientific) for 15 min at 95°C, followed by 40 cycles of 15 s at 95°C, 30 s at 60°C and 30 s at 72°C. *β-actin* was used as the housekeeping gene for nuclear and mitochondrial genes. Relative expression compared to controls was calculated using the ΔΔCt method (43).

### DNA isolation [genomic (gDNA) and mitochondrial (mtDNA)] and relative mitochondrial DNA content determination

HepG2 cells were harvested and cell pellets were snap-frozen in liquid nitrogen for storage. Cell lysis was performed overnight at 55°C in TNE lysis buffer (10 mmol/L Tris/HCl, pH 7.5; 150 mmol/L NaCl; 10 mmol/L EDTA; 1% SDS) and 100 μg Proteinase K. Total genomic cellular DNA, including nuclear and mitochondrial DNA, was extracted using chloroform/isoamyl alcohol (24:1), treated with RNase A (Thermo Scientific) for 1 h at 37°C, and then precipitated using isopropanol (also for Supplementary Figure S2). DNA concentrations were quantified using a Nanodrop 1000 spectrophotometer (Thermo Scientific, USA). Human and mouse mtDNA relative content was determined by qPCR using primers designed for CYTB versus β-actin and COX2 versus RSP18, respectively (44) (Supplementary Table S2). Mitochondrial DNA (Supplementary Figure S2) was isolated with the Mitochondrial DNA isolation kit (Abcam, Cambridge, UK), according to the manufacturer’s instructions.

Frozen human and mouse liver samples were homogenized using a pestle tissue grinder. Total DNA and RNA were extracted using the AllPrep DNA/RNA/Protein Mini Kit (Qiagen, Hilden, Germany) according to the manufacturer’s protocol. Mitochondrial DNA was isolated with the Mitochondrial DNA isolation kit (Abcam, Cambridge, UK), according to the manufacturer’s instructions. The DNA and RNA concentrations in each sample were measured by the NanoDrop 1000 spectrophotometer (Thermo Scientific) or Qubit.

### Pyrosequencing and sample preparation

One μg of DNA was first linearized using *Bam*HI (*fast digest*, Thermo Scientific) at 37°C for 1 h. 500 ng of DNA was then bisulfite-converted using the EZ DNA methylation Gold Kit (Zymo Research, Irvine, CA, USA) according to the manufacturer’s instructions. For pyrosequencing, bisulfite PCR of the *CSBII/III*, *COX1, D-loop*, *HSP1*, *LSP,* and *ND6* regions was conducted using bisulfite-specific primers (Supplementary Table S3). Primers were designed using the PyroMark Assay Design 2.0 software (Qiagen). To avoid amplification of NUMTs, primers designed for pyrosequencing analysis were evaluated *via* sequence homology analysis using BLASTn^1^ against the nuclear reference genome (Human or Mouse). In case of a highly probable nDNA hit, adjacent sequences (Additive sliding windows by 25 nucleotides up to 150) were separately run (BLASTn) and aligned against reference mtDNA and the nuclear genome to discard NUMTs. Only primers without strong hits in nuclear DNA were used. The PCR product was then run on a 2% agarose gel to confirm amplification and predicted amplicon length. The PCR product was then sequenced using the Q48 automated pyrosequencing machine (Qiagen) according to the manufacturer’s instructions. The percentage methylation at each CpN site was determined using the PyroMark Q48 Autoprep 2.4.2 software (Qiagen).

### Nanopore sequencing and mtDNA methylation analysis

DNA was extracted using Qiagen Blood Tissue DNA isolation kit (Cat no: 69504) without any modifications to the protocol. The quality of the extracted DNA was measured using Qubit (Thermo Fisher) for concentration, Little Lunatic (Unchained Labs) for purity and Fragment Analyzer (DNF-492 Large Fragment kit, Agilent) for integrity. Fragment Analyzer 5200 (Agilent Technologies). Assay kit used is either “Agilent DNF-464 HS Large Fragment Kit” (integrity of extracted hmw-DNA) or “Agilent DNF-492 Large Fragment Kit” (fragmentation and size selection). After the QC, 5 μg of DNA was fragmented using Megaruptor 3 (Diagenode) to final fragment sizing 15-20 kb, which resulted also in linearization of mtDNA and exposing fragments’ ends for end repair and adapter ligation. After Fragmentation, small molecules were depleted using Short Read Eliminator kit (SRE XS, PacBio). Short DNA fragments <10 kb were progressively depleted. DNA <4 kb is nearly completely removed.

Library preparation was started with 175fmol size selected DNA per sample (+/−2 μg of fragmented, size selected DNA). Barcoding kit EXP-NBD114 Native Barcoding Expansion 13–24 (PCR-free) (Oxford Nanopore Technologies) was used. Sequencing motor protein coupled to the specific barcoding adapter is the same as used in SQK-LSK109 (XL is the “high throughput”-version). Prior to the final sequencing adapter ligation, samples were pooled equimolar for optimal read distribution. Sequencing was performed on the R9.4.1 PromethION Flow Cell that had 8,750 pores available for sequencing. In total 50fmol of final library was loaded on the Flow cell (~550 ng). Total sequencing time was 80 h on PromethION 24 (Oxford Nanopore Technologies), with a flush using DNase I before loading of fresh library at 24 and 48 h of sequencing. mtDNA is well covered performing shallow gDNA sequencing (60–80× vs. 1× for gDNA). Reads were basecalled using GUPPY (version 6.0.6). Analysis was performed using a pipeline integrated in genomecomb (45). Reads were aligned to the hg38 genome reference (46) using minimap2 (47) and the resulting sam file was sorted and converted to bam using samtools (48). For methylation calls nanopolish (49) was used. The resulting sample sets of different individuals were combined and annotated using genomecomb (45).

Nanopolish analysis (version 0.13.2) was performed for CpG and GpC types (49) both for nuclear and mitochondrial genomes without applying NUMT filtering, as NUMTs were shown to only have a marginal impact on methylation assessment (49, 50). The sequencing run produced 12.49 M reads with an N50 of 17.39 kb, resulting in a total base output of 153.16 Gb in total data produced 1.2 TB.

### Methylation-specific PCR

Bisulfite-converted DNA was used as the template for a methylation-specific polymerase chain reaction (MSP). Two previously reported pairs of primers were used (8), that is, one pair specific for bisulfite-converted methylated DNA (M primers) (Sigma-Aldrich) and the other pair specific for bisulfite-converted unmethylated DNA (U primers). Each qPCR reaction contained 10 μmol/L of the antisense and sense primers (M/U primers) (Supplementary Table S4), 5 ng DNA and 2× ABsolute QPCR SYBR Green Rox Mix (Thermo Scientific). PCR was carried out on the ViiA7 Real-time PCR system (Applied Biosystems, Thermo Fisher Scientific) for 15 min at 95°C, followed by 40 cycles of 15 s at 95°C, 30 s at 60°C and 30 s at 72°C. Results were presented as ratios of CT values obtained for M primers vs. U primers normalized against CT values for U primers targeting the D-loop. The resulting ratios were expressed as methylated DNA vs. unmethylated DNA, as reported previously (8).

### Microscopy

To visualize mitochondria, an antibody against the mitochondrial protein MnSOD (manganese superoxide dismutase) was used. HepG2 wild-type cells and transgenic derivatives were cultured on glass coverslips for 24 h. At the termination of the experiment, cells were washed with ice-cold PBS and fixed with 4% paraformaldehyde (PFA) (Merck Millipore, Darmstadt, Germany) for 10 min. Cells were permeabilized using 0.1% Triton-X100 for 10 min at room temperature. Blocking was performed with 2% BSA for 30 min and cells were incubated with an anti-MnSOD2 antibody (Enzo Life Sciences, Brussels, Belgium) at a dilution of 1:1,000 for 1 h. Cells were washed and incubated with the secondary antibody, goat-anti-rabbit Alexa-488 (Invitrogen by Thermo Scientific, Waltham, USA). Coverslips were mounted onto glass slides using Vectashield mounting medium with DAPI (Vector Laboratories, Inc., Peterborough, UK) and fluorescence was visualized using a Leica DMI6000B inverted microscope (DFC365 FX camera) (Leica Microsystems, Wetzlar, Germany).

### Statistics

Cell line data are expressed as the mean ± SEM of at least 3 independent experiments, unless indicated differently, with three sets of the three transgenic cell lines created at three independent time points. Statistical analysis was performed using Graph-pad Prism 7 software. Single group comparisons were performed with the two-tailed unpaired student’s t-test. Human and mouse data were analyzed using a two-tailed Mann Whitney U test. Correlation analysis was conducted using Spearman’s correlation test and *p* values ≤0.05 were considered significant. As in this study, our comparative statistical analysis of cytosine methylation percentages to evaluate pyrosequencing data was hypothesis-driven, we did not adjust our significance levels for multiple testing, as was also previously suggested by others (51). A value of *p* < 0.05 was considered statistically significant (*p ≤ 0.05, **p < 0.01 and ***p < 0.001). For nanopore sequencing, the KNIME platform [version 4.6.1 (52)] was used to analyze chromosome-wide CpG or GpC methylation differences between the four stable cell lines for chrM and chr21, statistical data visualization was performed using Seaborn (53).

## Results

### Enhanced methylation of mtDNA in transgenic HepG2 cells expressing mtM.CviPI or mtM.SssI

**Figure 1:**
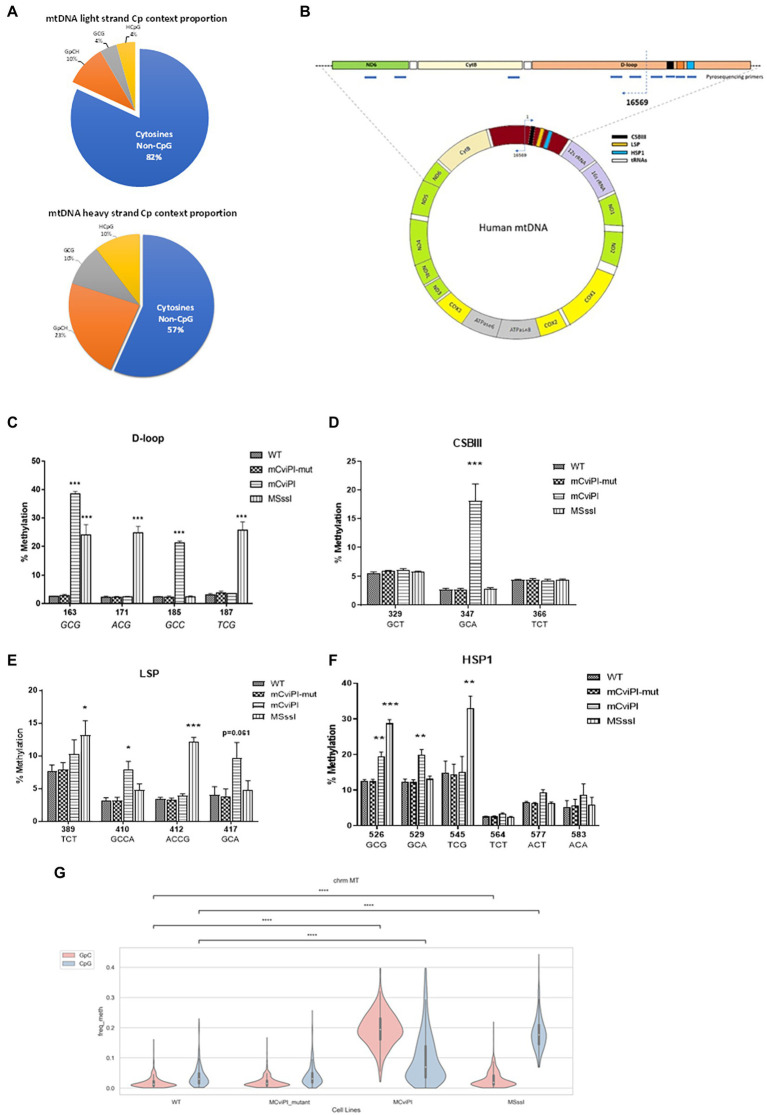
Methylation patterns in HepG2 cells expressing mitochondria-targeted methyltransferases (mtM.CviPI or mtM.SssI). **(A)** Pies representing the CpG/GpC distribution of cytosine residues in mtDNA. The blue portion represents all cytosines not in CpG context per strand. **(B)** Annotated human mitochondrial DNA showing pyrosequencing regions analyzed in this study; **(C)** *D-loop* (160–190); **(D)** *CSBIII* (320–370); **(E)** *LSP* (380–430) and; **(F)** *HSP* (525–585); Each data point represents the mean ± SEM of three independently constructed transgenic cell and *p* values as *p ≤ 0.05, **p < 0.01 and ***p < 0.001 with respect to the mtM.CviPI-Mut control. **(G)** Validation of differential mitochondrial genome methylation using nanopore long read sequencing. The mitochondrial genome was covered a 100%; >600-fold in all the four cell lines (****p < 0.0001).

Figure 1A shows a general overview of the distribution of cytosine residues in the human mtDNA of which the light strand (LS) contains 5,181 cytosines and the heavy strand (HS) 2,169 cytosines. Of these cytosines, 939 are in the symmetric GC and/or CG context. Pyrosequencing of DNA (at various regions, Figure 1B) derived from mitochondria-targeted mtM.CviPI or mtM.SssI-expressing HepG2 cells validated that the mitochondria-targeted methyltransferases induced methylation of mtDNA in the expected GpC and CpG context, respectively (Figures 1C–F). The highest degree of induced GpC methylation was observed for the targeted cytosine at position 163 and ranged from 2.5% in wildtype to 37.9% for mtM.CviPI, while the highest CpG methylation level was achieved for cytosine at position 545 (from 14.8% in wildtype to 33% in mtM.SssI expressing cells). The cytosine at GpC/CpG position 526 is targeted by both enzymes and resulted in 19.4 ± 2.6% methylation for mtM.CviPI versus 28.7 ± 2.0% for mtM.SssI (Figure 1F), while the GpC/CpG at position 163 showed 37.9 ± 1.5% methylation for mtM.CviPI versus 24.0 ± 3.6% for mtM.SssI (Figure 1C). Interestingly, some cytosines were resistant to induced methylation like the cytosine at position 329 (GpC) within the conserved sequence block 3 (CSBIII) which was resistant to mtM.CviPI-induced methylation (Figure 1D), while the cytosine at position 389 (TCT) was methylated by mtM.SssI (Figure 1E).

The methylation percentages for all cytosines measured in the wildtype HepG2 cells by pyrosequencing resulted in baseline readings ranging from about 2%, which did not further decrease after digesting the mtDNA at three positions using HindIII, as compared to a single cut with BamHI (Supplementary Figures S2A,B). For instance, CpG position 545, which is 16 bp away from the HSP1 transcription start site had a high percentage of both induced (33.0 ± 3.5 for HepG2-mtM.SssI), as well as baseline methylation (15.1 ± 4.3% for HepG2-mtM.CviPI) and controls: 14.9 ± 3.3% (HepG2-wt), 14.4 ± 2.8% (HepG2-mtM.CviPI-Mut) (Figure 1F). Also, no difference was observed for different isolation methods (total genomic DNA versus mtDNA, Supplementary Figure S2C). Analyzing mitochondrial genome cytosines upon bisulfite-independent nanopore sequencing validated similar GpC methylation in HepG2 wildtype and mtM.CviPI-mut cells. Although an increase in GpC methylation was also observed for mtM.SssI (mCpG) cells, which might be explained by cytosines in CpGpC contexts (Figure 1G), a clear increase in GpC methylation is found for mtM.CviPI cells. For CpG methylation, increased methylation was confirmed for mtM.SssI cells, with again some methylation induced in mt.MCviPI cells.

Importantly, all experiments were performed soon after creation of the transgenic cells as after 2 months in culture, the levels of induced mtDNA methylation were decreased (Supplementary Figure S3A), suggesting a silencing of the integrated expression cassette and/or a growth disadvantage due to mtDNA methylation. Nanopore sequencing, analyzed for off-target cytosines in the context of CpG and GpC on chromosome 21, showed no clear difference in methylation distribution patterns among HepG2 cell lines, although overall coverage was low (Supplementary Figure S3B). As only indivisible increases were detected for the very low GpC methylation levels, these findings confirmed that the methyltransferases were preferentially targeted to the mitochondria and not the nucleus (17).

### CpG or GpC methylation of mtDNA downregulates the expression of mitochondrial genes in HepG2 cells

**Figure 2:**
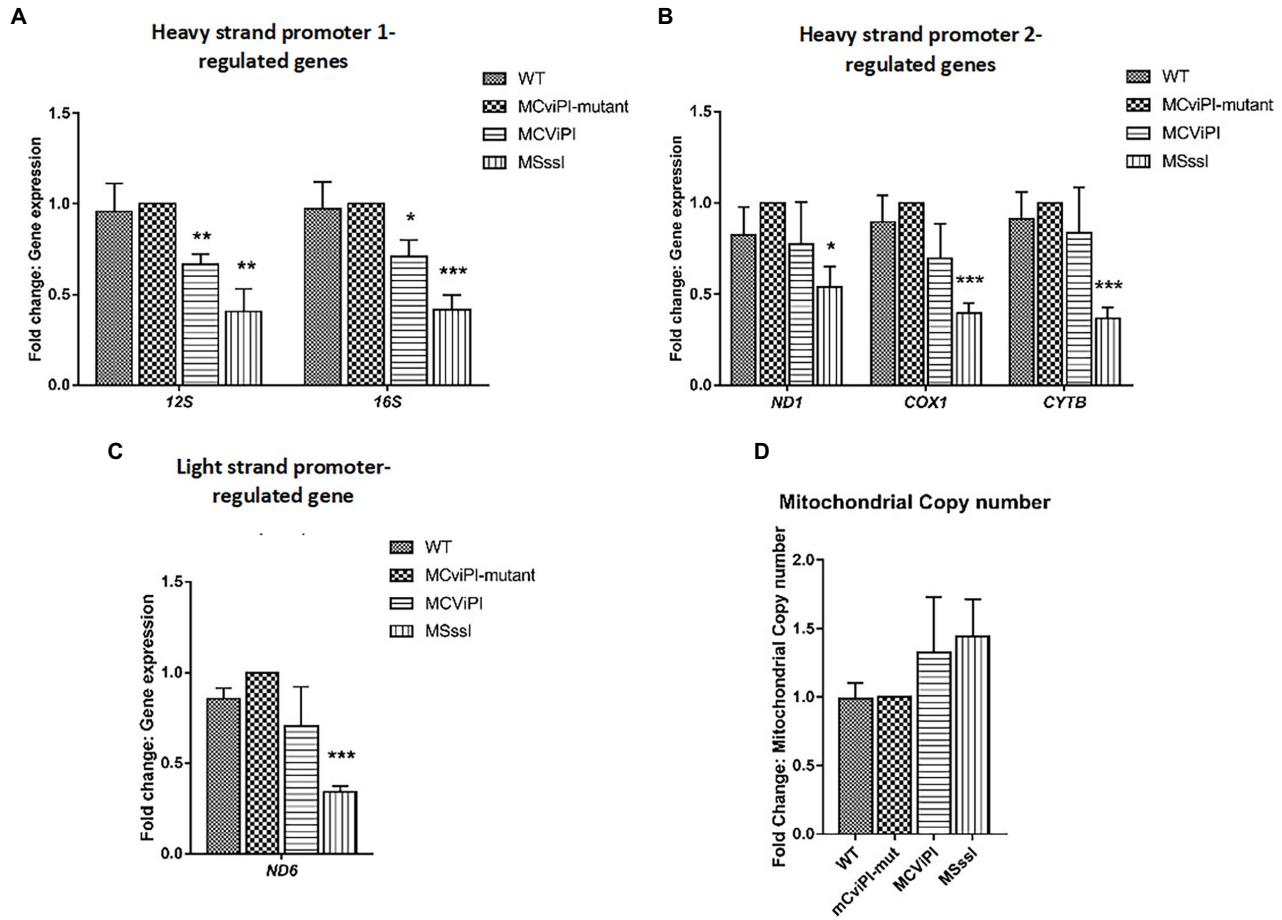
Normalized mitochondrial gene expression and mtDNA content in transgenic HepG2 lines expressing mitochondria targeted methyltransferases “mitochondrial DNA content” (M.CviPI or M.SssI). Expression of **(A)** *HSP1*; **(B)** *HSP2*; and **(C)** *LSP* genes normalized against HepG2-mtM.CviPI mutant control. **(D)** Mitochondrial DNA content in HepG2-mtM.CviPI and mtM.SssI normalized against mutant control. Each data point represents the mean ± SEM of three independently constructed clones per transgenic cell line. Significance is demonstrated as *p ≤ 0.05, **p < 0.01 and ***p < 0.001 with respect to the M.CviPI-Mut control.

Previously, we reported that artificially-induced GpC methylation of the mtDNA repressed expression of certain mitochondrial genes depending on the cell type (17). To determine the effects of mtDNA methylation on mitochondrial gene expression in the liver context, RT-qPCR was carried out on HepG2-mtM.CviPI, its mutant control and HepG2*-*mtM.SssI-expressing cells, as well as HepG2 wild type cells. No significant differences in mitochondrial gene expression were observed between wild-type cells and HepG2-mtM.CviPI-Mut controls for *HSP1* (*12S, 16S*)-, *HSP2* (*ND1, COX1, CYTB*)-, and *LSP* (*ND6*)-controlled genes (Figures 2A–C). In HepG2-mtM.CviPI cells, the *12S* and *16S* RNA levels were significantly reduced compared to HepG2-mtM.CviPI-Mut cells (66.6 ± 5.8% *p* < 0.01 and 71.0 ± 9.1%, *p* < 0.05, respectively) (Figure 2A). No clear difference in gene expression was observed due to the induced GpC methylation of the other genes tested. Interestingly, expression of all analyzed mitochondrial genes (*12S*, *16S, ND1, COX1, CYTB* and *ND6*) was decreased over 50% in HepG2-mtM.SssI, when compared to the mutant and parental control cells (Figures 2A–C). The lower gene expression could not be explained by a lower mtDNA content, as these levels were not significantly different between the analyzed cell lines (Figure 2D). Actually, when gene expression was normalized to mtDNA content for each sample, an even more pronounced lowering in expression was observed (Supplementary Figure S4). These data show that induced methylation in the GpC context, and even more obvious for the CpG context, decreased mitochondrial gene expression, without significant differences in mtDNA content in HepG2 cells.

### mtDNA methylation impairs lipid metabolism

**Figure 3:**
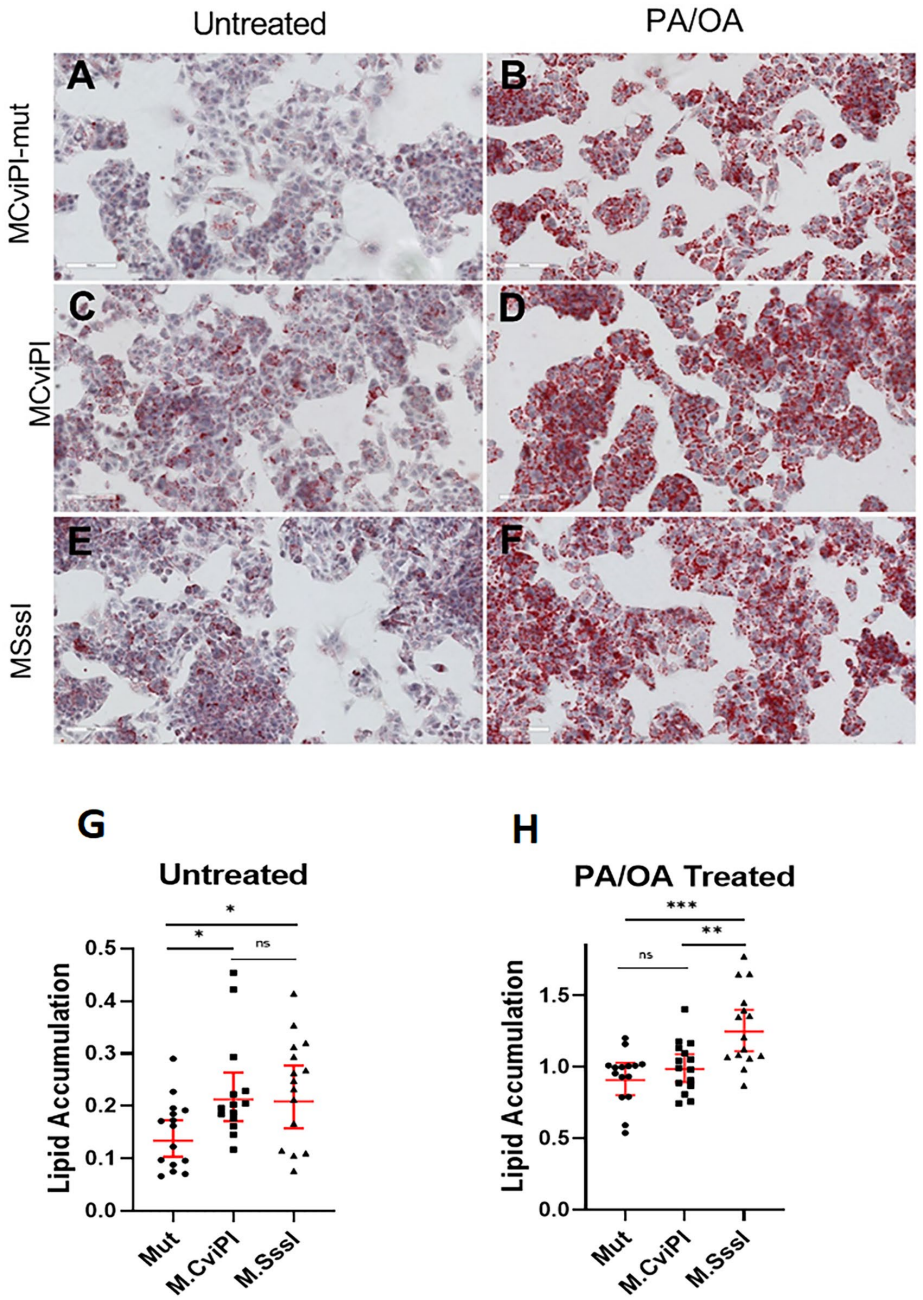
Oil Red O staining in HepG2 cells expressing mitochondria targeted methyltransferases (mtM.CviPI or mtM.SssI) after treatment with free fatty acids (PA/OA) (1 mmol/L). Representative analysis and quantification of lipid accumulation in **(A)** HepG2-mtM.CviPI-Mut; **(B)** HepG2-M.CviPI-Mut treated with PA/OA; **(C)** HepG2-mtM.CviPI; **(D)** HepG2-mtM.CviPI treated with PA/OA; **(E)** HepG2-mtM.SssI; **(F)** HepG2-mtM.SssI treated with PA/OA; **(G)** Oil red O quantification for untreated cells **(A, C, E)**; **(H)** Oil red O quantification for PA/OA treated cells **(B, D, F)** using ImageJ software. Quantification data represent five randomly taken pictures to every condition (cell line and treatment) per experiment. *p ≤ 0.05, **p < 0.01, and ***p < 0.001 with respect to the PA/OA treated mtM.CviPI mutant control. Three independent experiments were conducted.

To assess whether the altered mtDNA methylation status caused impaired lipid metabolism, we measured lipid accumulation in the transgenic cell lines after 48 h exposure to free fatty acids. Interestingly, without additional lipids, HepG2 mtM.CviPI and mtM.SssI cells showed significant increased lipid accumulation as measured by Oil red O staining (Figures 3C,E vs. Figure 3A, and quantification in Figure 3G). After treatment with palmitic acid and oleic acid (1 mmol/L), HepG2-mtM.SssI showed significant increased lipid accumulation against HepG2 mtM.CviPI and HepG2 mtM.CviPI-Mut transgenic lines (Figure 3F, vs. Figures 3B,D, and quantification in Figure 3H). Taken together, these data indicate that increased methylation of mtDNA associates with increased lipid accumulation in HepG2 cells.

### Exposure to palmitic acid affects mitochondrial and nuclear gene expression but does not induce mtDNA methylation in WT HepG2 cells

**Figure 4:**
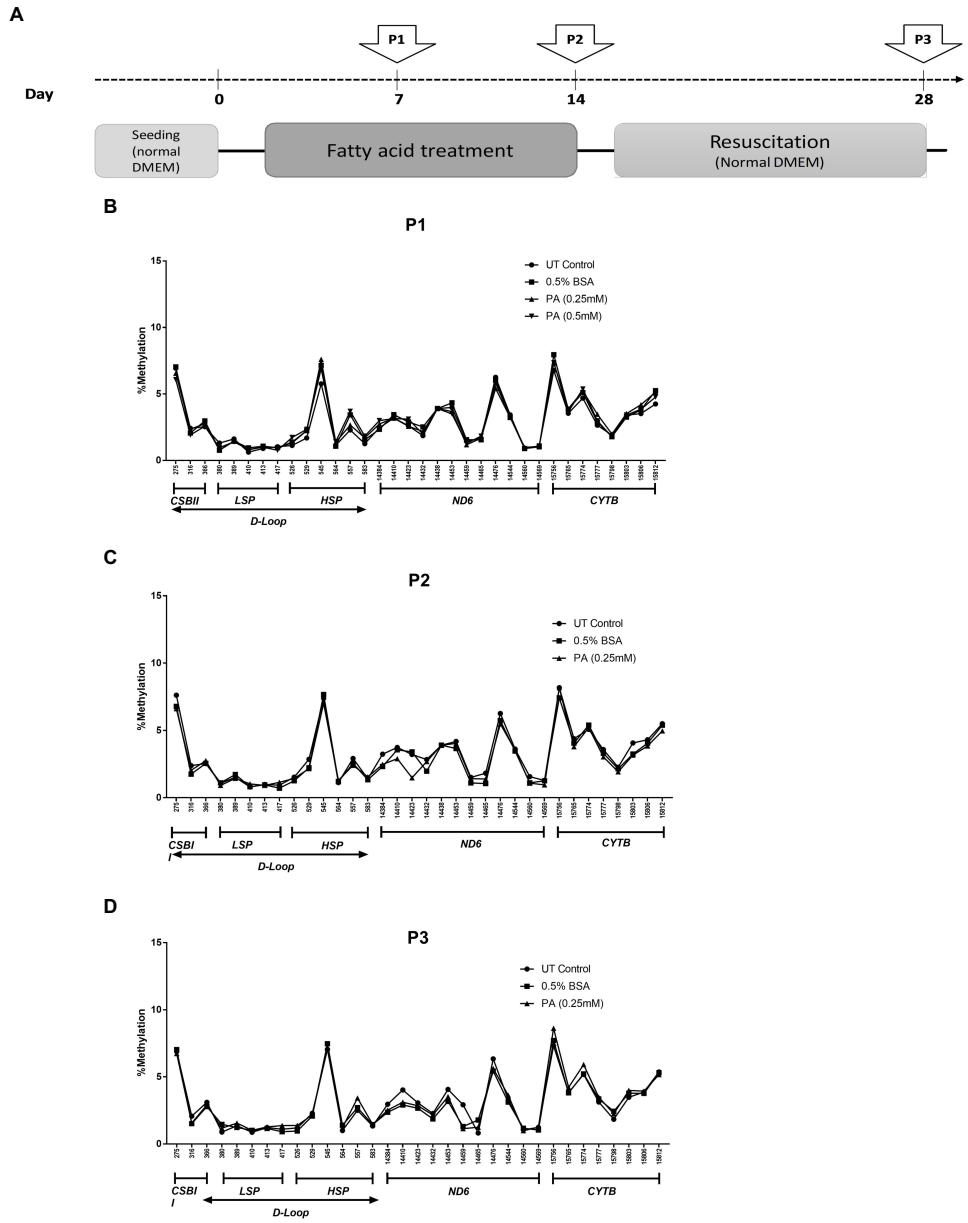
mtDNA methylation profile in HepG2 cells after long-term treatment with fatty acids [Palmitic Acid (PA)]. **(A)** Treatment scheme with arrows indicating the day of sampling (P1: day 7; P2: day 14; P3: day 28); **(B–D)** HepG2 cells treated with 0.25–0.5 mmol/L PA for 1 or 2 weeks (P1, P2) and; **(D)** HepG2 cells treated PA followed by an additional 2 weeks (P3) on resuscitation medium without PA (*n* = 1).

Saturated fatty acids are known to accumulate in hepatocytes during the progression of MeSH (54, 55) where they promote hepatic damage. In order to investigate whether mtDNA methylation can occur as a consequence of long-term lipid accumulation *in vitro*, wildtype HepG2 cells were exposed to palmitic acid (PA) for one or 2 weeks, and for 2 weeks followed by 2 weeks of recovery in normal medium without fatty acids (Figure 4A). Pyrosequencing resulted in highly reproducible patterns of mtDNA methylation in these HepG2 cell lines, ranging from 0.61 to 7.7% methylation at selected GpC and CpG sites, but neither PA treatment schedule induced any differential mtDNA methylation (Figures 4B–D).

**Figure 5:**
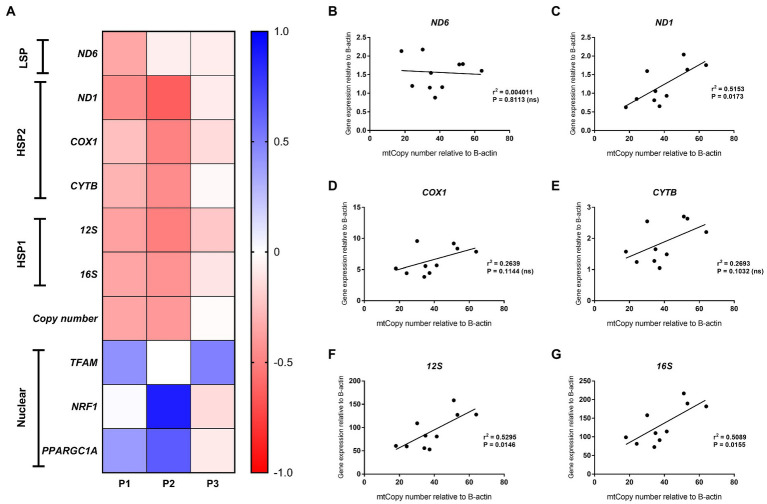
Mitochondrial and nuclear gene expression. **(A)** Heatmap of gene expression changes in HepG2 cells after treatment with 0.25 mmol/L palmitic acid (PA) for 1 or 2 weeks (P1, P2) and then an additional 2 weeks on resuscitation medium without PA (P3) (*n* = 1); **(B–G)** Correlation analysis of mitochondrial gene expression versus mtDNA content for HepG2 cells at P1 (treatment with 0.25 mmol/L and 0.5 mmol/L PA for 1 week), P2 (treatment with 0.25 mmol/L PA) and P3 [additional 2 weeks on resuscitation medium without PA for **(B)** *ND6*; **(C**) *ND1*; **(D)** *COX1*; **(E)** *CYTB*; **(F)** 1*2S*; and **(G)** *16S*].

Lowest methylation values were found for the *LSP* region (1.3%) compared to the *HSP* (2.7%), *ND6* (2.7%) and *CYTB* (4.1%) analyzed regions. The one or 2 week exposure of HepG2 cells to PA decreased mitochondrial gene expression (ranging from ~35% to 60%), but expression of all genes normalized after recovery of the cells in a medium without PA (Figure 5A). In contrast, *ND6* expression was reduced after 7 days of PA treatment but fully returned to normal after the prolonged PA exposure (P2) and remained relatively unchanged after the resuscitation phase when compared to the untreated HepG2 cells. In contrast, PA treatment induced the expression of nuclear genes involved in mitochondrial biogenesis (*TFAM, NRF1* and *PPARGC1A*) (Figure 5A). This effect was most pronounced at week 2 (P2), but was not associated with an increase in mtDNA content.

**Figure 6:**
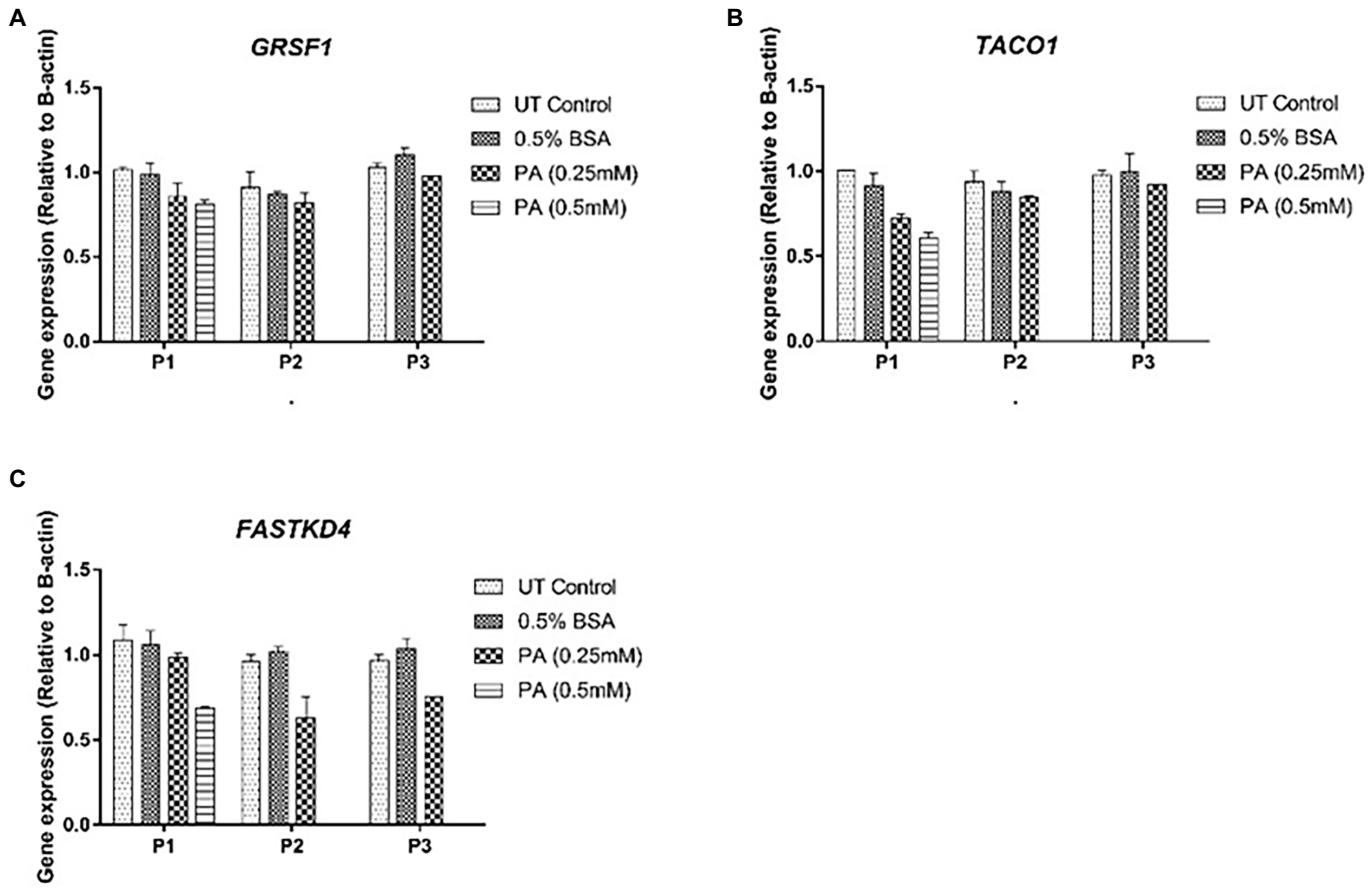
Gene expression profile of mtRNA binding proteins involved in mtRNA processing in HepG2 cells treated with 0.25 mmol/L palmitic acid (PA) for 1 or 2 weeks (P1, P2) followed by an additional 2 weeks (P3) on resuscitation medium without any PA (*n* = 1). **(A)** *GRSF1*; **(B)** *TACO1*; **(C)** *FASTKD4*.

Consistent with the generally accepted notion that mtDNA content correlates with gene expression, mitochondrial DNA content positively correlated with *ND1*, *12S* and *16S* gene expression (*r* = 0.72, *p* < 0.05, *r* = 0.73, *p* < 0.05 and *r* = 0.71, *p* < 0.05, respectively) for these treatment schedules. Intriguingly, no correlation was observed between *ND6*, *CYTB,* and *COX1* expression and relative mtDNA content (Figures 5B–G). These differences could be due to post-transcriptional mtRNA processing involving proteins like GRSF1, FASTKD4 and TACO1**.** Expression of *GRSF1* (responsible for *ND6* mRNA processing) and *TACO1* (affecting *COX1*), was not affected by PA treatment (Figures 6A,B). Interestingly, *FASTKD4* which affects the bulk of mtRNAs, was downregulated by PA treatment to about 60% and this downregulation was sustained after reculturing the cells in normal (PA-free) medium (Figure 6C). Overall, in this *in vitro* model of lipid-mediated cell stress, we did not observe that excessive cellular lipid accumulation modulates mtDNA methylation, although lipids affected (mitochondrial) gene expression levels (Figures 4B–D).

### Mice on a high fat diet show increased mtDNA methylation in the *Nd6* gene

**Figure 7:**
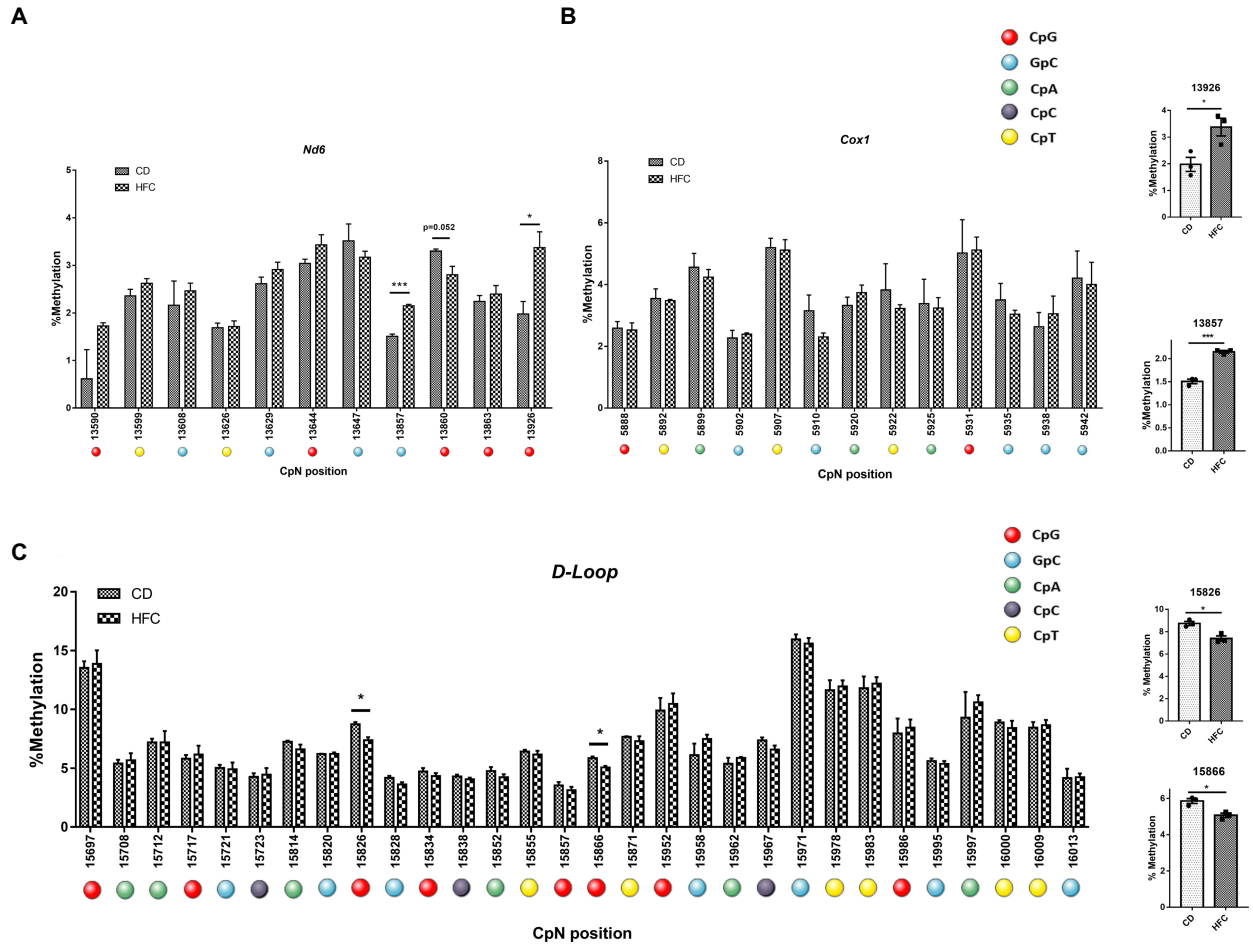
Pyrosequencing on whole mouse liver. MtDNA from mice on high fat and cholesterol diet (HFC) versus chow diet (CD) for 20 weeks was assessed for methylation. The analyzed regions include; **(A)** *Nd6*; **(B)** *COX1* and; **(C)** mtDNA *D-loop*. **#** dual GpCpG context. Data represent the mean ± SEM. *p ≤ 0.05, **p < 0.01, and ***p < 0.001 with respect to the chow control (*n* = 3 vs. 3). The individual data points per animal for the four statistically significant different cytosines are shown separately for **(D)** Nd6 and **(E)** Dloop.

Since no clear induction of mtDNA methylation was observed in PA-exposed HepG2 cells, we assessed whether the hypermethylation of *ND6* reported for MeSH (8) could be explained by the *in vivo* context of inflammation and fibrosis. In order to study this, mice were fed a high fat-high cholesterol diet (HFC) for 20 weeks to mimic advanced stages resembling MeSH (lipid accumulation associated with inflammation and fibrosis; 20wkHFC), as described earlier (41, 42). Induction of fibrosis in the 20wkHFC model was confirmed by increased hepatic expression of fibrotic markers, *Col1a1* and *Acta2* (54, 56). mtDNA was pyrosequenced for the *D-loop*, *Cox1* and *Nd6* regions for these 20wkHFC-fed mice and normal chow-fed mice. Interestingly, in line with the previous findings in humans, significant increases in methylation were observed in the *Nd6* gene in mice at positions 13,857 (*p* < 0.001) and 13,926 (*p* < 0.05) (Figure 7A) compared to the control-fed mice. No differential methylation was observed in the *Cox1* gene for 20wkHFC mice compared to the controls (Figure 7B). Methylation within the D-loop region was lower at two CpG positions (15,826 and 15,866; p < 0.05) for 20wkHFC-fed mice compared to the control-fed animals (Figure 7C).

**Figure 8:**
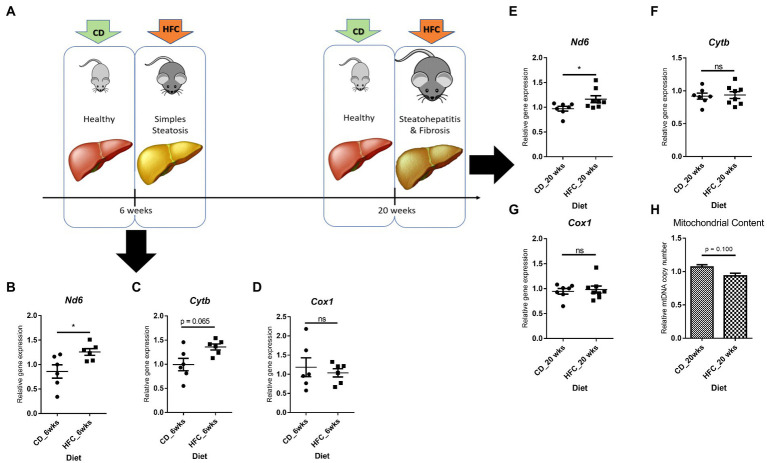
Gene expression of three mitochondrial genes and mitochondrial DNA content in mice on high fat and cholesterol diet (HFC) versus chow diet (CD) for 6 weeks and 20 weeks. **(A)** Schematic diagram showing experimental set-up. Mitochondrial gene expression in CD versus HFC after 6 weeks: **(B)** *Nd6*; **(C)** *CytB*; **(D)** *Cox1*. Mitochondrial gene expression in CD versus HFC after 6 weeks: **(E)** *Nd6*; **(F)** *CytB*; **(G)** *Cox1*; **(H)** Mitochondrial copy number. Data represent the mean ± SEM. *p ≤ 0.05, **p < 0.01 and ***p < 0.001 with respect to the CD control animals (*n* = 6 vs. 6).

Interestingly, *Nd6* expression was significantly increased (p < 0.05) in these mice, as well as in mice mimicking early stages of MAFLD (lipid accumulation and mild inflammation, without fibrosis; 6wkHFC) when compared to control-fed animals (Figures 8A,B,E). Inflammation and fibrosis markers were not increased in 6wkHFC mice compared to control-fed animals (57), confirming that the disease state indeed had not yet progressed to MeSH. *CytB* expression also showed a trend toward increased expression (*p* = 0.065), but only in 6wkHFC mice, while no change in *Cox1* expression was observed at either time point (Figures 8C,D,F,G). Changes in gene expression were not related to changes in copy number as determined for the 20wkHFC mice (Figure 8H). Expression levels of mitochondrial genes were compared for both models (Supplementary Figure S5A–F) and found to be similar in both 6-week and 20-week control-fed groups. The increase in expression of *Nd6* was also similar for the 6wkHFC versus the 20wkHFC mice (Supplementary Figure S5D), and the unresponsiveness in the expression of *Cox1* was seen for both models (Supplementary Figure S5E).

### Human liver samples

**Figure 9:**
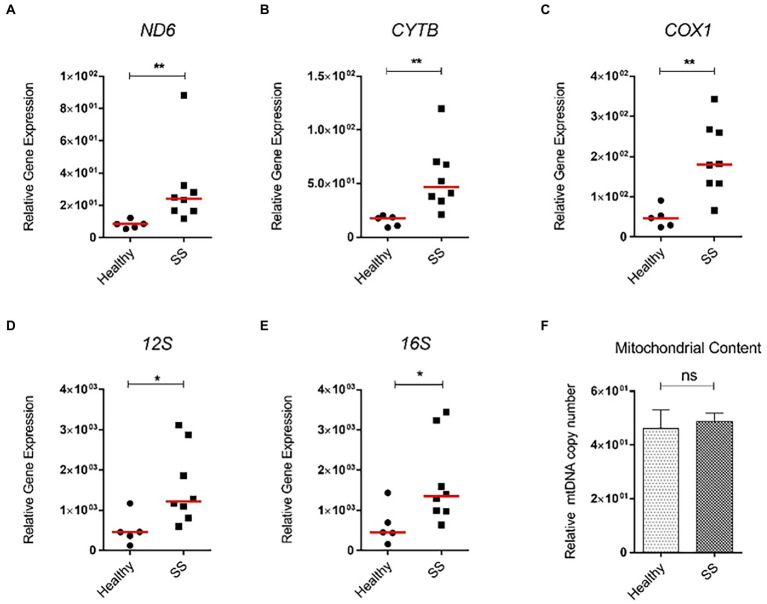
Relative mitochondrial gene expression and mtDNA content in whole human liver samples from obese bariatric surgery patients (with SS). Human liver samples from bariatric surgery patients were obtained. These liver samples had characteristics of SS. mRNA expression of **(A)** *ND6*; **(B)** *COX1*; **(C)** *CYTB*; **(D)** *12S*; **(E)** *16S* genes, and **(F)** mitochondrial DNA content. All relative to *β-actin* mRNA or DNA. Red line represents the median. *p ≤ 0.05, **p < 0.01, and ***p < 0.001 with respect to the control.

Next, we analyzed steatotic liver samples from morbid obese individuals who underwent bariatric surgery and compared those to non-steatotic human liver tissue. While hepatic mRNA levels of *PNPLA3*, a biomarker of MAFLD, were significantly enhanced in steatotic liver tissue when compared to non-steatotic human liver, expression of inflammatory (*TNFα, IL1β*) and fibrotic markers (*COL1A1, ACTA2*) in these patients was not increased (data not shown). Interestingly, similar to the mice, *ND6* expression was significantly higher in steatotic livers (*p* < 0.01) compared to the healthy controls (Figures 9A,B). Other mitochondrial genes, such as *CYTB, COX1*, *12S* and *16S,* were also significantly elevated (*p* < 0.05) compared to the healthy controls (Figures 9C–E). Intriguingly, the overall increase in gene expression was not associated with higher mtDNA content between steatotic and non-steatotic human livers (Figure 9F).

**Figure 10:**
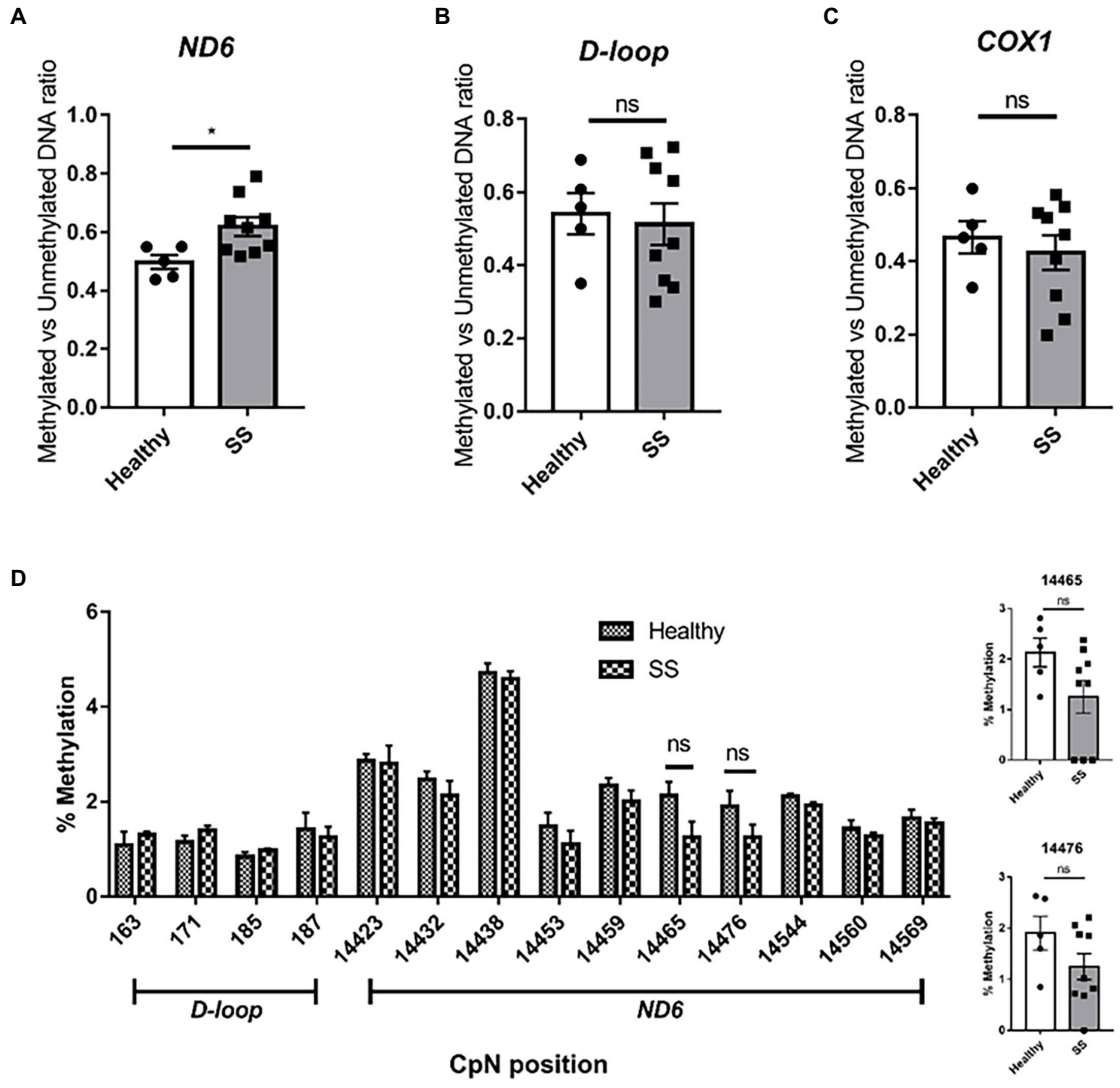
Methylation specific PCR (MSP) and pyrosequencing on whole human liver samples from obese bariatric surgery patients (with SS). Methylation Specific PCR on **(A)** *ND6*; **(B)** *D-loop*; **(C)** *COX1* on healthy controls (*n* = 5) versus SS patients (*n* = 9); **(D)** *D-loop* [163–187] and *ND6* [14423–14569] pyrosequencing on healthy controls versus SS patients. Significance is demonstrated as *p ≤ 0.05, **p < 0.01, and ***p < 0.001 with respect to the healthy controls.

Using the previously described primers for methylation-specific PCR (8), we confirmed the increase in *ND6* methylation in steatotic livers compared to non-steatotic livers (methylated/unmethylated DNA ratio of 0.62 and 0.50, respectively; *p* < 0.05) (Figure 10A). No changes in methylation were found for the *D-loop* and the *COX1* gene (Figures 10B,C). Surprisingly, for *ND6*, no increases in methylation were observed among the interrogated cytosines by pyrosequencing analysis (Figure 10D).

## Discussion

We here addressed the potential role for *ND6* mtDNA methylation in MAFLD, also based on earlier reports describing that *ND6* methylation was higher, and associated with lower *ND6* expression in (1) liver samples of MeSH patients compared to samples obtained from SS patients (8), and (2) leucocytes of Type II diabetes patients (22). Our studies in patient liver samples also point to a role for mtDNA methylation in MAFLD as we confirmed differential mtDNA methylation in the *ND6* gene for SS patients compared to controls, using the same approach (MSP) described earlier by Pirola et al. (8) and Cao et al. (22). Unfortunately, our pyrosequencing analysis did not pinpoint cytosines with a higher *ND6* methylation level. As shown in Supplementary Figure S6, this seemingly discrepancy might be explained in terms of MSP primers coverage that interrogate only three CpG positions (not included in our pyrosequencing analysis), two in the forward and one in the reverse primers. Further analysis should include a more extended methylation analysis at the *ND6* region for a better understanding of its biological meaning. In addition to the earlier study (8), we found the *ND6* expression to be higher for SS samples compared to healthy samples, pointing to mitochondrial compensation mechanisms to be associated with MAFLD progression from healthy to SS (higher *ND6* expression) to MeSH (decreased *ND6* expression) (8). To provide mechanistic insights, we exposed liver cells to fatty acids, created transgenic liver cell lines and additionally analyzed two stages of MAFLD in mice. We demonstrate that (i) mtDNA methylation decreased overall mitochondrial gene expression, which (ii) promoted lipid accumulation, while (iii) long-term *in vitro* lipid exposure did not induce mtDNA methylation, but high-fat high cholesterol diet did affect *in vivo* mtDNA methylation.

The lack of effect on mtDNA methylation in our 2 weeks PA exposure study using HepG2 cells excludes a causal role for PA on mtDNA methylation. Other dietary lipids, however, still need to be investigated for their effects, as olive oil-based regimens did induce differential hepatic mtDNA methylation in fish (after 70 days of administration), whereas PA indeed did not show methylation differences with respect to the controls (58). Interestingly, using a higher PA concentration for a shorter time period (3 mmol/L, 24 h), *Cao* and coworkers reported increased methylation of *ND6* by MSP in HepG2 cells which could be functionally linked to AMPK-induced translocation of DNMT1 to the mitochondria (22). Despite the lack of effect on mtDNA methylation measured by pyrosequencing, our PA treatment did modulate (mitochondrial) gene expression levels, including an upregulation of nuclear *PPARGC1A* encoding PGC1α, probably indicating increased fatty acid oxidation. In another study, 48 h PA exposure of muscle cells resulted in a downregulation of nuclear *PPARGC1A* expression, which impaired mitochondrial biogenesis (59).

Also steatotic (16 samples) and MeSH (7 samples) patients showed reduced expression of nuclear-encoded mitochondrial proteins (PGC1α, NRF1 and TFAM) compared to lean controls, while mitochondrial proteins that constitute complex I, II, IV and V of the ETC was reduced only in MeSH cases (60). Although we did not find reduced expression of the nuclear genes involved in mitochondrial biogenesis, mtDNA content and mtDNA gene expression generally was repressed by PA. Interestingly, despite a lower mtDNA content, *ND6* expression was restored during the second week of PA treatment. This relative increase in expression of *ND6* compared to other mitochondrially encoded genes is in line with the increase observed for *Nd6* expression and not for *CoxI,* in our HFC mouse models. While a decrease in *Nd6* expression was observed for HFD mice livers by Cao et al. (22), their *in silico* analysis indicated an increased *ND6* expression in livers and not in most other somatic tissues of Type 2 diabetes patients.

Also, an increase in *ND6* expression was observed in our SS patient samples compared to healthy liver samples. These findings on increased liver *ND6* expression seem in contrast to the earlier human MAFLD studies (8), which reported a decreased liver *ND6* expression in MeSH compared to SS liver samples. In this respect, it is important to note that we normalized the expression data against *β-actin*, and not against *12S or 16S,* as done by others (8), which we here and previously (17) found to be regulated by mtDNA methylation. Yet, the initial *ND6* increase as reported by us for SS, confirmed for Type 2 diabetes patients (22), followed by a decrease in *ND6* expression when progressing to MeSH (8), would fit the proposed compensation model of dynamic regulation as a response to mitochondrial dysfunction during disease progression (10, 11, 14).

Mitochondrial DNA methylation would add an additional layer to such dynamic regulation processes. Indeed, we did confirm increased *ND6* methylation in MAFLD both in mice using pyrosequencing as well as in human tissue using the previously reported CpG-focused MSP approach (8, 22). However, in our study, pyrosequencing analysis did not confirm a higher *ND6* methylation in human tissue. This discrepancy might be explained by various technical reasons as the technologies cannot be compared in a simple, straightforward manner: while pyrosequencing assesses individual cytosines (not only in CpG context), MSP data only reflect the combined methylation status of three CpGs targeted by the primers [covering regions 14245 to 14269 (forward) and 14459 to 14487 (reverse); see Supplementary Figure S6]. Although our pyrosequencing primers could be designed to address two closely neighboring regions (14384–14476 and 14544–14569), the two approaches do not interrogate the same cytosines. The fact that differential *ND6* methylation has been demonstrated by MSP in two other independent studies (8, 22) indicates that more detailed investigations are warranted. Yet, the indirect influence of DNA methylation (e.g., through inhibition of DNMT1) on *ND6* expression has been indicated by others (8, 61, 62). As *ND6* expression is under the control of the LSP promoter, the inverse relationship between LSP promoter methylation and *ND6* expression, as supported by our transgenic HepG2 cells and confirmed by others (26), should be explored in more detail for MAFLD samples. Since mtDNA, a supercoiled, protein-fixed structure with likely non-CpG methylation resulting in strand-specific patterns, is different from nuclear DNA, different considerations compared to nuclear DNA might apply (26, 32, 63). Understanding mtDNA methylation might eventually lead to the exploitation of this additional layer of regulation. In this respect, it is essential to mention that data by us and others based on bisulfite conversion might not reflect actual DNA methylation *per se* but can also reflect hydroxymethylation and observed differences might even indicate differences in bisulfite accessibility. Regarding hydroxymethylation, Pirola and co-workers analyzed overall levels of 5hmC in fresh liver samples from MAFLD patients at different stages (13). Using immuno-specific assays, the authors did not detect significant differences between MAFLD samples and near-normal controls. Nevertheless, patients with MAFLD displayed a significant loss in non-nuclear 5hmC staining, which might reflect an overall loss of mitochondrial genome hydroxymethylation. As hydroxymethylation has been reported to also occur on mitochondrial genomes (23), further research into this epigenetic mark is warranted. Moreover, available data based on bisulfite conversion might not fully reflect actual DNA methylation but rather indicate differences in bisulfite accessibility. Indeed, the current debate is based on the mtDNA coiled structure inducing bisulfite resistance, which leads to mtDNA methylation overestimation. In this respect, we recently described an analysis using LC–MS/MS (24) and showed that for mtDNA isolated from the TriZol RNA fraction (with very low levels of nuclear DNA contamination), cytosine methylation levels were below our current detection limit in 5 out of 9 samples tested (0.2%). Noteworthy, such findings do not exclude the existence of biologically relevant cytosine methylation in certain regions. In the current study, therefore we determined mtDNA methylation using another bisulfite independent approach (nanopore sequencing) and could clearly validate the differential methylation patterns of the different transgenic HepG2 cell lines we created. Moreover, our nanopore sequencing data of wildtype HepG2 provide further indications of (be it low-level) CpG methylation on the mitochondrial genome. Our analyses of GpC methylation did not support high-level of non-CpG methylation on mitochondrial DNA (26), and as such is in line with a recent paper challenging this notion (64). Improved long read sequencing analysis algorithms will shed light on the ongoing debate on the existence of endogenous mtDNA methylation (34–37).

In conclusion, we confirm that artificially-methylated mtDNA promotes mitochondrial dysfunction (17) and disturbs the cellular lipid metabolism in the liver context. In addition, our findings support a role for mtDNA methylation (or other parameters affecting bisulfite resistance) in MAFLD progression. However, kinetics during disease progression (8) and as a response to external factors (22) need to be further studied to understand the dynamic nature of mtDNA responses better.

As epigenetic changes are reversible, these can be targeted for therapeutic interventions, e.g., by epigenetic editing strategies (65–67). A better understanding of mtDNA methylation (and/or other parameters explaining the bisulfite resistance) might thus allow for innovative treatment options. Indeed, DNA-targeted approaches are currently explored to remove mutated mitochondrial DNA from diseased cells (68). To modulate DNA methylation, DNMT and TET enzymes can be targeted to loci of interest already reaching mainstream applications for nuclear DNA (69). Since DNA (de)methylating enzymes also localize to mitochondria (70), these approaches might turn out effective in treating mitochondrial dysfunction for a range of diseases.

## Data availability statement

The original contributions presented in the study are publicly available. This data can be found here: https://www.ncbi.nlm.nih.gov/, BioProject (PRJNA956894).

## Ethics statement

The animal study was reviewed and all procedures were approved by the Ethics Committee (Institutional Review Board) of the University Hospital Essen (Reference Number: 09–4252) and the study protocol conformed to the ethical guidelines of the Declaration of Helsinki. The patients/participants provided their written informed consent to participate in this study. The animal study was reviewed and approved by All procedures were approved by the Landesamt für Natur-, Umwelt-, und Verbraucherschutz Northrhine Westfalia (LANUV NRW) and the Landesverwaltungsamt Saxony-Anhalt (reference number: 84.0204.2013.A082).

## Author contributions

AM, KF, and MR: conceived the study. AM, FC-M, and JH: experiments. BS, SS, and LB: *in vivo* analysis. VM: patients recruitment and human samples. IAI, PdR, TdP, WB, and CT: nanopore sequencing. AM, FC-M, KF, and MR: wrote the manuscript in collaboration with all authors. All authors contributed to the article and approved the submitted version.

## Funding

AM was funded through a talent development program at the UMCG. FC-M was funded by the Colombian Ministry of Science (Minciencias, COLCIENCIAS-COLFUTURO № 783–2017) and Instituto Tecnológico Metropolitano, Medellin. This project was funded by De Cock-Hadders initiative (Project code WB-11/2021).

## Conflict of interest

The authors declare that the research was conducted in the absence of any commercial or financial relationships that could be construed as a potential conflict of interest.

## Publisher’s note

All claims expressed in this article are solely those of the authors and do not necessarily represent those of their affiliated organizations, or those of the publisher, the editors and the reviewers. Any product that may be evaluated in this article, or claim that may be made by its manufacturer, is not guaranteed or endorsed by the publisher.

## Footnotes

^1^ https://blast.ncbi.nlm.nih.gov/Blast
