# Supplementary material for: Mitochondrial DNA methylation in metabolic associated fatty liver disease
Source: Front Nutr. 2023 May 25;10:964337. doi: 10.3389/fnut.2023.964337 (PMC10249072; doi:10.3389/fnut.2023.964337)
Supplement: Supplementary file 1 [file Data_Sheet_1.docx]

| **Plasmid** | **Quantity (µg)** |
| --- | --- |
| Vector | 3 |
| Gag/pol/rev | 2 |
| Envelope | 1 |

**Supplementary Table S1.** Lentiviral transfection plasmid DNA ratios

| **Target** | **Forward primer 5′→3′** | **Reverse primer 5′→3′** |
| --- | --- | --- |
| ND1 | ATACCCCCGATTCCGCTACGAC | GTTTGAGGGGGAATGCTGGAG |
| ND6 | GGGTGGTGGTTGTGGTAAAC | CCCCGAGCAATCTCAATTAC |
| COX1 | CGATGCATACACCACATGAA | AGCGAAGGCTTCTCAAATCA |
| CYTB | AATTCTCCGATCCGTCCCTA | GGAGGATGGGGATTATTGCT |
| 12S | CTGCTCGCCAGAACACTACG | TGAGCAAGAGGTGGTGAGGT |
| 16S | GTATGAATGGCTCCACGAGG | GGTCTTCTCGTCTTGCTGTG |
| PGC1a | TGAGAGGGCCAAGCAAAG | ATAAATCACACGGCGCTCTT |
| NRF1 | GGGAGCTACAGTCACTATGG | TCCAGTAAGTGCTCCGAC |
| TFAM | CCGAGGTGGTTTTCATCTGT | TCCGCCCTATAAGCATCTTG |
| β‐actin | CCAACCGCGAGAAGATGA | CCAGAGGCGTACAGGGATA |
| ND6 (M) | GTTGGAGTTATGTTGGAAGGAG | CAAAGATCACCCAGCTACTACC |
| COX1 (M) | CCCAGATATAGCATTCCCACG | ACTGTTCATCCTGTTCCTGC |
| CYTB (M) | CCCACCCCATATTAAACCCG | GAGGTATGAAGGAAAGGTATAAGGG |
| 36B4 (M) | GCTTCATTGTGGGAGCAGACA | CATGGTGTTCTTGCCCATCAG |
| COX2 (M-dna) | ATAACCGAGTCGTTCTGCCAAT | TTTCAGAGCATTGGCCATAGAA |
| RSP18 (M-dna) | TGTGTTAGGGGACTGGTGGACA | CATCACCCACTTACCCCCAAAA |

**Supplementary Table S2.** Primers for mitochondrial DNA gene expression and relative content

| **Region** | **Strand** | **Target Location** | **Sequences 5′→3′** |
| --- | --- | --- | --- |
| D-loop (H) | HS | 16412 - 16457 | **Fw:** GGGTTATTTAGGTTTTATGATTTTGAAG  **Rv:** ATAACACATTACAATCAAATCCCTTCTC  **Seq:** GTTTATTTTAGTTATTTTTAAGTGT |
| D-loop (H) | HS | 16084 - 16131 | **Fw:** GGTTGATTGTTGTATTTGTTTGTAAGT  **Rv:** CACCATTAACACCCAAAACTAAAATTCTA  **Seq:** TTTATGTATTATAGGTGGTTAAG |
| D-loop (H) | HS | 163 - 190 | **Fw:** GTTTGGTGGAAATTTTTTGTTATGATGT  **Rv:** CTTTAATTCCTACCTCATCCTATTATTT  **Seq:** AATTAATATATTTTAGTAAGTATG |
| CYTB (H) | HS | 15756 - 15812 | **Fw:** TTAATTAGGGAGATAGTTGGTATTAGGA  **Rv:** CAATAATCCCCATCCTCCATATATCC  **Seq:** AGGATTGTTGTGAAGT |
| CSBII (H) | HS | 275 - 316 | **Fw:** GGAGGGGAAAATAATGTGTTAGT  **Rv:** CCACTTTCCACACAAACATCATA  **Seq:** TTTAAGTGTTGTGGTTAGA |
| CSBIII (H) | HS | 329 - 366 | **Fw:** GGAGTGGGAGGGGAAAAT  **Rv:** CTCCCCCCCTTCTAACCACAAC  **Seq:** TGGTTAGGTTGGTGT |
| HSP (H) | HS | 526 - 583 | **Fw:**  AGTGTATTGTTTTGAGGAGGTAAG  **Rv:**  ACCCCCCAACTAACACATTATT  **Seq:**  GTTTTGAGGAGGTAAGTT |
| LSP (H) | HS | 366 - 417 | **Fw:** GAGTGGGAGGGGAAAATAATGTGTTA  **Rv:**  AACCACAACACTTAAACACATCTCTA  **Seq:**  GTTGGGGGGTGATTG |
| ND6 (H) | HS | 14544 - 14569 | **Fw:** GGGTTTGTGGGGTTTTTTTTTAAG  **Rv:**  TTAAACCCATATAACCTCCCCCAAAATTC  **Seq:**  TTTTATTTATGGGGGTTTAG |
| ND6 (H) | HS | 14384 - 14476 | **Fw:**  GTGGTAGGGTGTGTTATTATTTTGAATT  **Rv:**  ACCACCCCATCATACTCT  **Seq:**  GATGGTTGTTTTTGGATA |
| COX1 (M) | LS | 5888 - 5942 | **Fw:**  GTTGGAGTGTTATTTATTTTAGGTGTAAT  **Rv:**  AAAATTAAATCCCCTCCTCCA  **Seq:**  ATTAAATTTTATTATTTGTTTGAT |
| D-loop (M) | LS | 15697 - 15723 | **Fw:** TGTTATAAGGATATATTTGTGTTATTTGA  **Rv:** ATTTCAATTTAACTACCCCCAAATTT  **Seq:** TTATTTGGTTTATTAATTTATTATT |
| D-loop (M) | LS | 15820 - 15871 | **Fw:** GTTTATTAAATTTGGGGGTAGTTAAATTGA  **Rv:** AAATACCAAATACATAACACCACAAT  **Seq:** ATTTGGTTTTTATTTTAGGGTT |
| D-loop (M) | LS | 15952 - 16013 | **Fw:** GGAGAGTTAAAATTTGGTATTGAGTAGT  **Rv:** ATCAACCCATAACCAACATAACTATAA  **Seq:** GTTTTAGGTGATTGGG |
| ND6 (M) | LS | 13590 - 13647 | **Fw:**  TTGGGAGATTGGTTGATGTATGA  **Rv:**  CTTTATATCATTCCTAATTAACATCATCTT  **Seq:**  GTTATGTTGGAAGGAGG |
| ND6 (M) | LS | 13857 - 13926 | **Fw:**  GTGGGTTTGTTGGTTGTTTAATG  **Rv:**  CCCCAAATCTCTAAATATTCCTCAA  **Seq:**  TTAGGGTTTGGTGGA |

**Supplementary Table S3.** Pyrosequencing primers and design template**. (H):** Human**, (M):** Mice**, D-loop**: Displacement loop, **CYTB**: Cytochrome B, **CSBII/III**: Conserved sequence block 2/3, **HSP**: Heavy strand promoter, **LSP**: Light strand promoter, **ND6**: NADH-ubiquinone oxidoreductase subunit 6.

|  |  | **The primers sequences for methylation-specific PCR** | |
| --- | --- | --- | --- |
| **Gene** | **Location** | **Forward primer 5′→3′** | **Reverse primer 5′→3′** |
| ND6-M | 14242 - 14484 | TTTCGTATTAATAGGATTTTTTCGA | AATTATCTTTAAATATACTACAACGAT |
| ND6-U | 14242 - 14484 | TTTTGTATTAATAGGATTTTTTTGA | ATAATTATCTTTAAATATACTACAACAAT |
| COX1-M | 5948 - 6151 | GGAATATTATATTTATTATTCGGCGT | ACTAATCAATTACCAAAACCTCCG |
| COX1-U | 5948 - 6151 | TGGAATATTATATTTATTATTTGGTGT | CTAATCAATTACCAAAACCTCCAAT |
| D-loop-M | 38 - 144 | TAGGAATTAAAGATAGATATTGCGA | ACTCTCCATACATTTAATATTTTCGTC |
| D-loop-U | 38 - 144 | GGTAGGAATTAAAGATAGATATTGTGA | ACTCTCCATACATTTAATATTTTCATC |

**Supplementary Table S4.** Methylation Specific PCR primers. ***M**, methylated-specific primers; **U**, unmethylated-specific primers (8)
