# Supplementary material for: Mitochondrial DNA methylation in metabolic associated fatty liver disease
Source: Front Nutr. 2023 May 25;10:964337. doi: 10.3389/fnut.2023.964337 (PMC10249072; doi:10.3389/fnut.2023.964337)
Supplement: Supplementary file 2 [file Data_Sheet_2.docx]

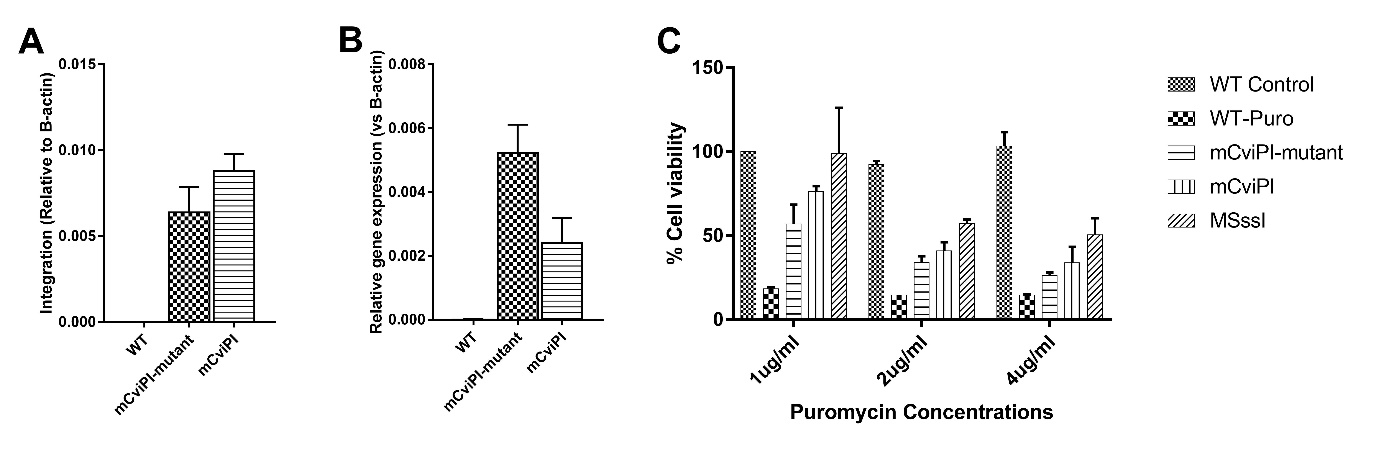


**Supplementary Figure 1**. **Validation of transgenic HepG2 cell lines.** **A-B**) Integration and gene expression of mtM*.*CviPI

in HepG2 cells; **C)** Puromycin selection of stable HepG2 cell line expressing mtM.CviPI, mtM.CviPI-Mut (mutant) and mtM.SssI

versus HepG2-WT Control (no puromycin) and WT-puro control (puromycin added).

**
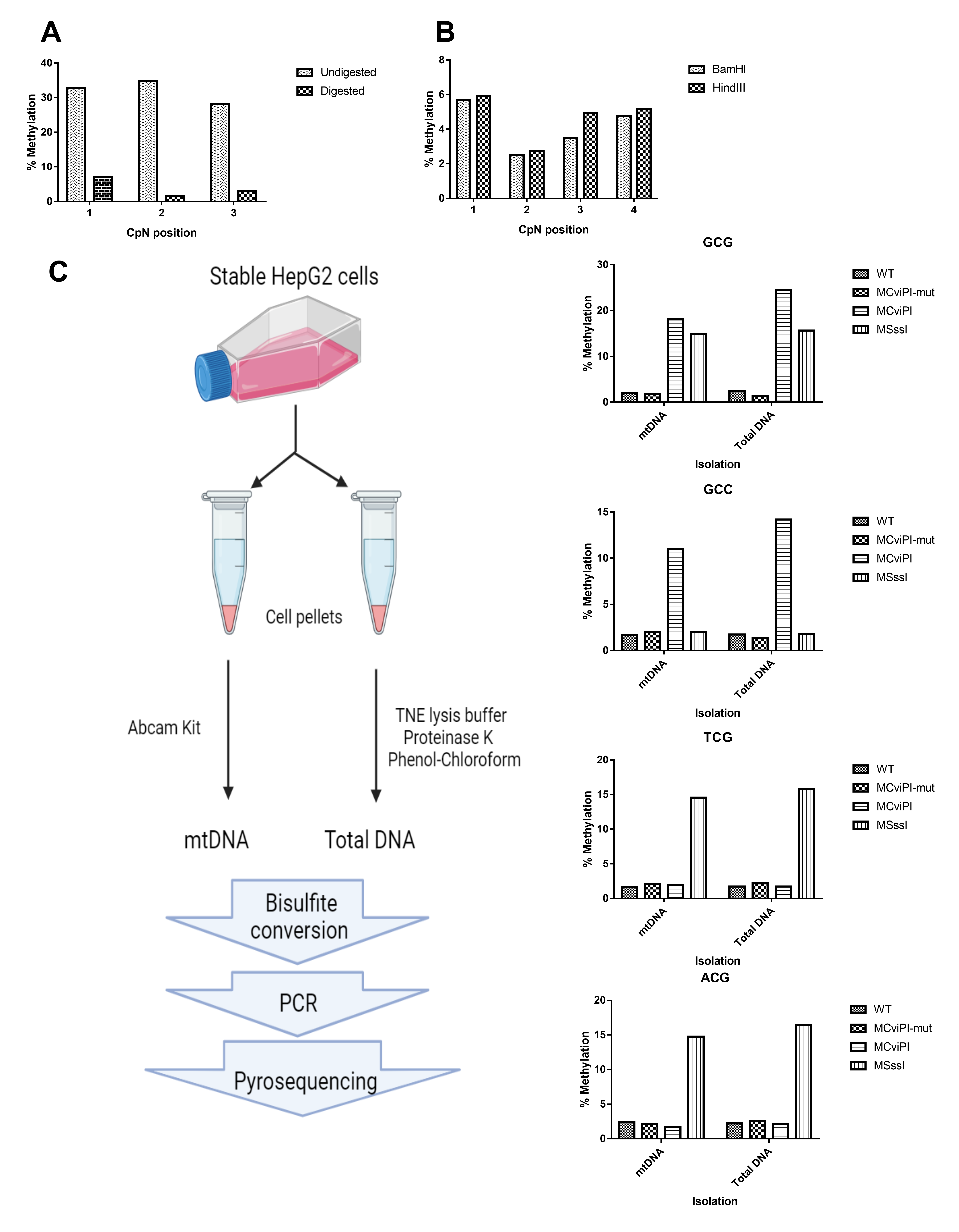
**

**Supplementary Figure 2.** MtDNA linearization and pyrosequencing of total genomic DNA versus mtDNA. A) Undigested versus *Bam*HI digested DNA; B) *Bam*HI versus *Hind*III digestion; C) Total genomic DNA versus mitochondrial DNA at CpG/GpC positions in HepG2 (wild type) and transgenic HepG2 lines expressing mitochondria targeted methyltransferases (mtM.CviPI or mtM.SssI).

**
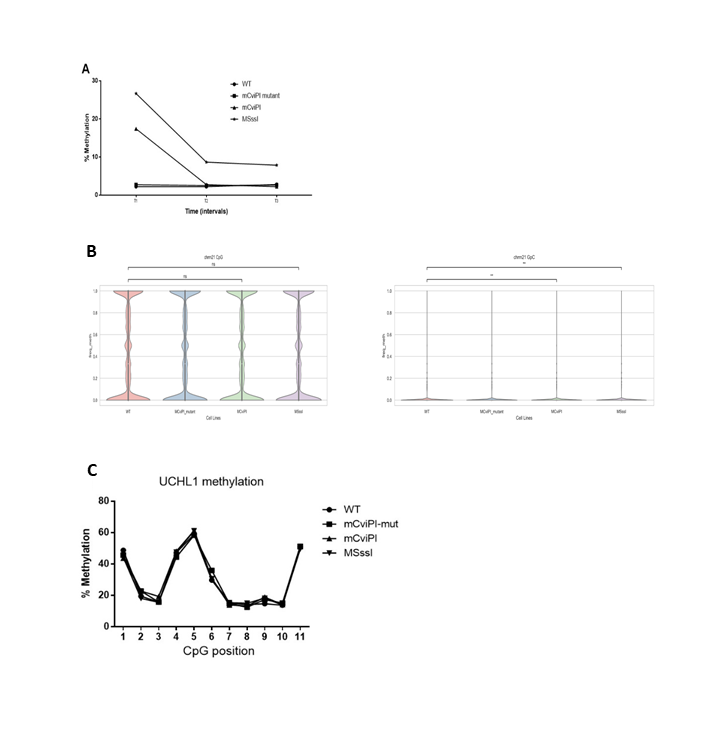
**

**Supplementary Figure 3: Characterization of transgenic cell lines. A) kinetics of** mtDNA methylation in HepG2 cells expressing *mtM.SssI and mtM.CviPI* over a 3-month period (T1 – T3); **B)** Off-target comparative methylation plotted as violin plots for all cytosines in CpG (left violins) or GpC (right violins) context of Chromosome 21. Overall similar patterns were obtained for DNA isolated from wild type HepG2 cells, as for transgenic HepG2 cells expressing mitochondrial targeted mtM.CviPI-mut, mt M.CviPI and mtM.SssI).**C)** *UCHL1* promoter region methylation did not show differences for the different cell lines.


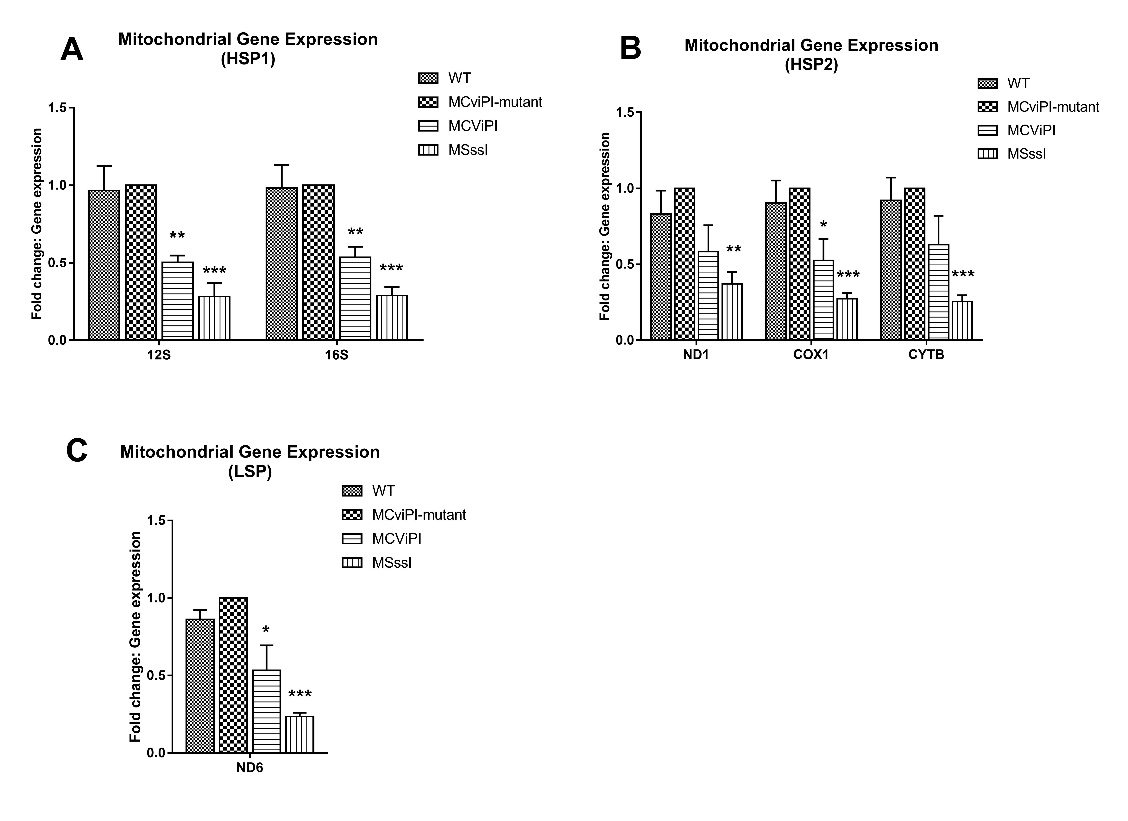


**Supplementary Figure 4.** **Mitochondrial gene expression normalized to mtDNA relative content** **in transgenic HepG2 lines expressing mitochondria targeted methyltransferases (mt*M.CviPI* or mt*M.SssI*)**. Expression of A) *HSP1*; B) *HSP2* and; C) *LSP* genes normalized against HepG2-mt*M.CviPI*-Mut control and mtDNA relative content. Each data point represents the mean ± SEM of three independently constructed clones per transgenic cell line. Significance is demonstrated as *p≤0.05, **p<0.01 and ***p<0.001 with respect to the mt*M.CviPI*-Mut control.


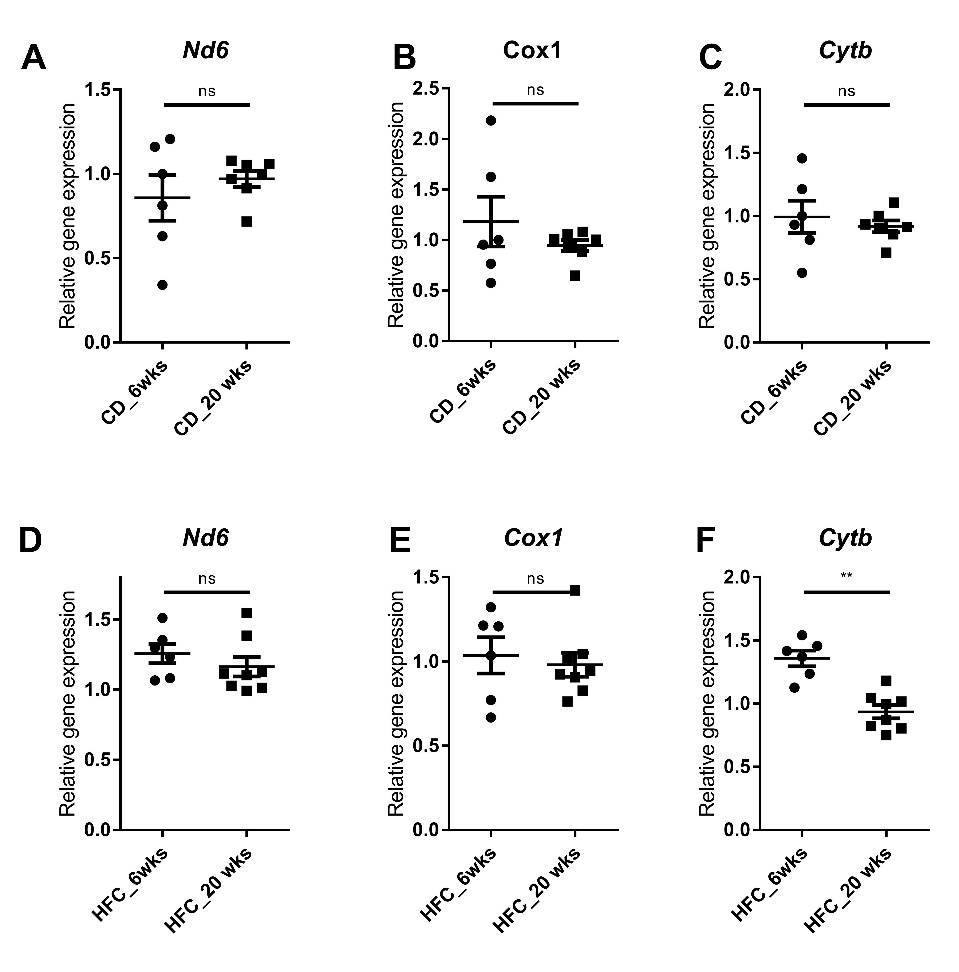


**Supplementary Figure 5: Comparison of gene expression of three mitochondrial genes** in mice on chow diet (CD) for 6 weeks versus 20 weeks: A) *Nd6*; B) *Cox1;* C) *CytB* and in mice on high fat and cholesterol diet (HFC) for 6 weeks versus 20 weeks: D) *Nd6*; E) *Cox1;* F) *CytB.* Data represent the mean ± SEM. *p≤0.05, **p<0.01 and ***p<0.001 with respect to the CD control animals.

**Supplementary Figure 6: Locations of primers for Methylation Specific PCR (MSP) and for pyrosequencing of *ND6*.** Pairwise analysis was run (MEGA v11.0.13) including mitochondrial DNA light strand (reference genome NC012920) and aligned to the Forward and Reverse primers for Methylated (Methyl) and Unmethylated (UnM) sequence designed by *Pirola et al* (8). Numbers highlight nucleotide (nt) positions on mtDNA where encircled ones highlight MSP-investigated CpGs. Blue boxes indicate the pyrosequenced regions of the *ND6* gene (denoted in green color).
